# Identification and elimination of false positives in electrochemical nitrogen reduction studies

**DOI:** 10.1038/s41467-020-19130-z

**Published:** 2020-11-03

**Authors:** Jaecheol Choi, Bryan H. R. Suryanto, Dabin Wang, Hoang-Long Du, Rebecca Y. Hodgetts, Federico M. Ferrero Vallana, Douglas R. MacFarlane, Alexandr N. Simonov

**Affiliations:** 1 School of Chemistry, Monash University, Clayton, VIC 3800 Australia; 2 ARC Centre of Excellence for Electromaterials Science, School of Chemistry, Monash University, Clayton, VIC 3800 Australia

**Keywords:** Catalytic mechanisms, Electrocatalysis, Heterogeneous catalysis, Energy, Characterization and analytical techniques

## Abstract

Ammonia is of emerging interest as a liquefied, renewable-energy-sourced energy carrier for global use in the future. Electrochemical reduction of N_2_ (NRR) is widely recognised as an alternative to the traditional Haber–Bosch production process for ammonia. However, though the challenges of NRR experiments have become better understood, the reported rates are often too low to be convincing that reduction of the highly unreactive N_2_ molecule has actually been achieved. This perspective critically reassesses a wide range of the NRR reports, describes experimental case studies of potential origins of false-positives, and presents an updated, simplified experimental protocol dealing with the recently emerging issues.

## Abstract (web summary)

Discovering a sustainable route to ammonia as a fertiliser and as an energy carrier is critically important, but many recent reports on the electrochemical nitrogen reduction are false positives. Here the authors uncover the emerging experimental traps and detail protocols to reliably avoid them.

## Introduction

Ammonia is widely utilised in the agricultural sector as a source of fertilisers, and is now also recognised as a future carrier of renewable energy due to its large hydrogen content (17.6 wt%) and high energy density (4.32 kWh L^−1^ at −33.3 °C and 1 bar, 6.25 kWh kg^−1^). A recently published roadmap^1^ delves into the key aspects and anticipated stages of the development of the technologies that will underpin the *Ammonia Economy* in the coming decades. In this context, renewable energy-powered electrosynthesis of NH_3_ from nitrogen in the air and water is predicted to become a core technology.

The currently considered modes of the electrochemical conversion of N_2_ to NH_3_ can be tentatively separated into two major groups: (i) direct electrocatalytic reduction, and (ii) redox-mediated electroreduction^1^. Both can be further sub-categorised based on operating temperature, type of electrolyte/solvent system, and proton source used and on the basis of a range of other specific parameters pertinent to each mode, *e.g*. continuous *vs*. batch multistep process in mode (ii). Unsurprisingly, the most intense investigative attention has been devoted to the highly technologically attractive direct nitrogen reduction reaction (NRR) using aqueous electrolytes, which avoids the use of high temperatures, flammable and toxic solvents, and can theoretically operate at reasonable overpotentials^2^. Notwithstanding the truly significant effort invested in the development of this approach, the progress toward the demonstration of practical rates of NH_3_ production, that are well beyond background levels of nitrogen-containing contaminants, remains unconvincing.

Since 2010, a very broad range of materials has been reported to exhibit measurable and sometimes significant electrocatalytic activity for the NRR with aqueous electrolytes (Supplementary Table 1). The chemical and structural variety of these NRR “catalysts” ranging from trivial carbon-supported gold or nickel oxide particles to more exotic multicomponent materials are both impressive and puzzling—puzzling in the sense that according to these reports, the N_2_ molecule which traditionally is considered to be inert under ambient conditions, unexpectedly appears to be reasonably easy to activate, on a wide range of surfaces. In contrast to several recently published reviews providing only a perfunctory summary of the reported results on the NRR, we aim herein to critically revaluate the reliability of these reports and identify any work that qualifies as “genuine NRR”. We aim to highlight the level of experimental rigour in published papers as well as to point out opportunities for future improvement, whereby the research field can learn from this recent history to generate genuine progress.

To this end, in evaluating published NRR reports we have adopted three key criteria:

(I) Is the NH_3_ yield rate sufficiently high?
(II) Are the experiments using ^15^N_2_ reliable and sufficient to confirm the key results?
(III) Are the control and quantification of the oxidised forms of nitrogen that may be present in the experiments sufficiently rigorous?

Our literature summary on the aqueous NRR consists of 127 recently published papers up to April 2020 which is presented in Supplementary Table 1. In addition, we include a similar analysis of a range of publications on the electroreduction of N_2_ in organic media in Supplementary Table 2. Each criterion in these tables is colour-coded using a standard traffic lights scheme, *i.e. red* indicates that the criterion is not met, *orange*—possibly met or met in part, and *green*—sufficiently met. Further detailed explanations of these criteria are provided in the following section ‘Potential origins of false NRR positives’. Based on these criteria, we reach the unfortunate conclusion that none of the publications in Supplementary Table 1 qualifies for three *greens* and only one scores two *greens*, in **Criteria I** and **II**. However, our recent reassessment of the latter study suggests that even in this case no NRR actually occurs^3,4^. We elaborate on this example and similar false positives in other key material families in the section on ‘Case studies’, showing the strong significance of **Criterion III**, that has otherwise been largely overlooked to date. The organic media based reports, specifically those on the lithium-mediated process in Supplementary Table 2, fare somewhat better, allowing in some cases for a link-back to relevant experiments in previous work.

Thus, the question of the feasibility of the direct electrocatalytic reduction of N_2_ to ammonia with aqueous electrolytes remains open. To support meaningful progress in the field we reduce our findings into an updated protocol that aims to guide the researchers towards avoiding these issues. We also offer some comments on theoretical studies that are often used to justify otherwise unexpectedly positive results. We conclude with our thoughts on future directions that might prove fruitful in this vitally important field.

## Potential origins of false NRR positives

In what follows, we elaborate on each of the key reliability criteria introduced above and highlight the most common shortfalls in typical electrochemical NRR experiments.

### Criteria for reliable NRR data

(I) Sufficient ammonia yield rate (*v*/nmol s^−1^ cm^−2^_geom._)
  To enter the domain of applied significance, electroreduction of N_2_ should generate ammonia at a practically significant rate. The reported targets for *v* vary from 100 nmol s^−1^ cm^−2^ (Giddey et al.^5^) to *ca* 930 nmol s^−1^ cm^−2^ (US Department of Energy REFUEL program^6^). Although these estimates assume the use of high-surface area electrocatalyst layers in a final device, it should be noted that many of the NRR reports already rely on comparatively thick electrodes such as carbon fibre paper or various forms. In essence, this means that a normally expected possibility of increasing the laboratory research yield rate by at least an order of magnitude via integration of developed catalysts into industrial type electrodes is already a feature in many of NRR reports.
  In addition, the ammonia yield rate is a useful indicator of the reliability of the study. Even with the contemporary highly sensitive methods of analysis, quantification of the amounts of ammonia at levels that hardly exceed the level of the ubiquitous nitrogen-containing contaminants presents significant reliability challenges—reliable in the ultimate sense that the data can be reproduced in another laboratory.
  On the basis of these considerations, we have adopted the following scale for **Criterion I**:

**Table:** 

Red: Ambiguous and too low to be promising	*v* < 0.1 nmol s^−1^ cm^−2^
Orange: Plausible in principle yet unlikely to be practical	0.1 ≤ *v* < 10 nmol s^−1^ cm^−2^
Green: Highly plausible and promising	*v* ≥ 10 nmol s^−1^ cm^−2^
(II) Quantitative analysis of ^15^NH_3_ produced via electroreduction of ^15^N_2_
  Upon publication of several authoritative protocol papers on the NRR^7–9^, the majority of researchers have recognised the requirement for control experiments using ^15^N_2_ as the reactant, though very few implement those in a sufficiently reliable manner. Initially, the most common oversight was the use of only a qualitative mode of analysis of ^15^NH_3_ (*i.e*. no quantitative comparison is attempted with the main ^14^N_2_ data), which is entirely inconclusive in the NRR context. More recently emerging problems are associated with the misuse of the highly convenient method for the quantitative analysis of ^15^NH_4_^+^—proton nuclear magnetic resonance (^1^H NMR)^10^. To observe a clear ammonium triplet for ^14^N_2_ and doublet for ^15^N_2_, complete ammonia protonation must be achieved and be precisely controlled^10^, which is very rarely done. The most common fundamental mistake is quantitative analysis of spectra that are subject to a very significant and uncontrolled level of hydrogen/deuterium (H/D) exchange. Due to the rapid exchange of ammonium protons with a solution, it is imperative that the deuterated solvent does not contain any labile deuterium. Stable solvents such as DMSO-d_6_ and CDCl_3_ are recommended, and we stress that under no circumstances should D_2_O be used if the goal is to obtain a clear spectrum suitable for quantitative analysis. Finally, a single ^15^N_2_ experiment is not sufficient; the demonstration of ^15^NH_3_ generation over several experiment durations, at a rate and selectivity that match those measured for the ^14^N_2_ experiments under identical conditions, is recommended^11^.
  These considerations form the basis for the reliability scale for **Criterion II**:

**Table:** 

Red: Unreliable	Qualitative ^15^NH_3_ analysis only, or attempts of a quantitative ^15^NH_3_ analysis using inadequate experimental procedures (*e.g*. H/D exchange in NMR).
Yellow: Inconclusive, yet might be reliable	Reliable quantitative demonstration of the ^15^NH_3_ production rate and faradaic efficiency under one set of conditions only.
Green: Reliable	Quantitative ^15^N_2_ reduction data as a function of time provided in satisfactory agreement with corresponding key ^14^NRR data, based on rigorous and fundamentally sound procedures and methods.
(III) Control over the oxidised forms of nitrogen (NO_*x*_)

Notwithstanding being very well-known ubiquitous contaminants, that have been identified as the source of many false-positives in the biochemical N_2_ fixation field^12,13^, NO_*x*_ compounds remain strangely and largely ignored by the electrochemical NRR field. This point has been emphasised in the above-mentioned protocols^7–9^, and technical solutions to remove NO_*x*_ were proposed^14–17^. Considering this and the long history of diagnosing of the effects of ^14^NO_*x*_ and ^15^NO_*x*_ contaminants in biochemical N_2_ fixation studies, the following scale for **Criterion III** is applied in Supplementary Tables 1 and 2:

**Table:** 

Red: Insufficient control of NO_*x*_	No attempts to quantify NO_*x*_ in all key components.
Orange: Both N_2_ and NO_*x*_ reduction might occur	The amount of NO_*x*_ has not been fully quantified in the gas supplies and/or the electrocatalytic tests.
Green: NO_*x*_ reduction is likely to be negligible	The amount of NO_*x*_ has been quantified in gas supplies and in parallel with ammonia analysis in all key data on the reduction of both ^14^N_2_ and ^15^N_2_.

We note that the reliability-coding for **Criterion III** specifically emphasises *quantification* of NO_*x*_ compounds, rather than just providing measures for scavenging these contaminants. In other words, a statement on the implementation of measures to prevent contamination of an experiment with the oxidised forms of nitrogen, without reporting the actual amount of NO_*x*_ determined after control measures have been implemented, is not sufficient to prove the reliability of an NRR experiment. Since NO_*x*_ contaminants can enter experiments at any stage (even depending on atmospheric conditions) a completely sufficient basis for genuine NRR results must include determination of NO_*x*_ present in experimental samples at the same time as ammonia. In our opinion, the problem of NO_*x*_ contaminants is currently the most significant concern in the ammonia electrosynthesis field (Supplementary Tables 1 and 2 and refs. ^4,8,18^) and requires extended discussion, which follows below.

### Reduction of the oxidised forms of nitrogen *vs*. NRR

**Fig. 1:**
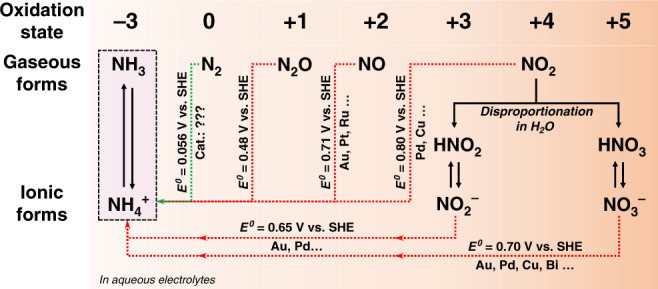
Electroreductive synthesis of ammonia. Pathways towards NH_3_/NH_4_^+^ through the electrochemical reduction of N_2_, gaseous NO_x_, and ionic NO_x_ compounds with standard redox potentials (vs. standard hydrogen electrode) and examples^27–30,54–57^ of some of the known electrocatalysts for each process.

Electroreduction of gaseous (N_2_O, NO, NO_2_) and ionic (NO_2_^−^ and NO_3_^−^) oxidised forms of nitrogen provides thermodynamically and kinetically more favourable pathways to ammonia than direct NRR (Fig. 1). Thus, genuine nitrogen electroreduction results can only be obtained when N_2_ is the only possible source of ammonia formation, *i.e*. all NO_*x*_ contaminants beyond the background are removed.

There are many possible sources of NO_*x*_ contaminants in an NRR experiment, though most of them pertain to the following three common scenarios. First, atmospheric NO_*x*_ compounds are ubiquitous in the laboratory and easily accumulate on various surfaces^9,19,20^, *e.g*. on glassware, gloves, chemicals, and electrodes (especially those with high surface area), as well as in solutions exposed to air^20^. Second, ionic NO_*x*_ impurities might be present in chemicals used as electrolytes^21^ or for the preparation of catalysts^18,22^. Third, the most insidious and hard to control are gaseous NO_*x*_ compounds inevitably contained in the N_2_ gas at even the highest levels of purity provided by the commercial suppliers. This problem can be exacerbated considerably by the variability of the actual purity of the gas supply from batch to batch.

Moreover, the majority of the available ^15^N_2_ gas supplies are even more contaminated with ^15^NO_*x*_ that are formed as by-products during the synthesis of ^15^N_2_ via the CuO-catalysed oxidation of ^15^NH_3_^23^. As such, ^15^NO_*x*_ concentrations can be as high as 0.1 mol% with respect to ^15^N_2_ and are highly variable, even when the gas comes from a reputable source^13^.

Traditionally, experiments investigating electrocatalytic reactions involving gaseous reactants/products like H_2_, O_2_, and CO_2_ are undertaken with a relevant gas being continuously bubbled through the electrolyte solution to ensure their saturation throughout measurements. This practice has been broadly adopted by the NRR field, although the rates of the reaction are so low that there is in fact no need for a continuous gas flow. Most importantly, the NO_*x*_ contaminants contained in the N_2_ source are continuously accumulated and reduced in such “flowing gas” experiments. Simple calculations (Table 1) suggest that the maximal possible ammonia formation yields and yield rates through the reduction of NO_*x*_ (assuming all contaminants are NO_*x*_ and are selectively reduced to NH_3_) at typical gas flow rates (20–100 mL min^−1^) can easily exceed those commonly reported for NRR experiments (Supplementary Tables 1 and 2). This problem is further aggravated if the N_2_ gas is purged with the bubbles hitting the electrode surface directly or with gas-diffusion electrodes, as these configurations substantially facilitate the direct adsorption of NO_*x*_ onto the electrode surface.

**Table 1:** Amount of N_2_ and potential NO_x_ contaminants supplied in 4-h^a^ NRR experiments undertaken at different gas supply rates.

Flow rate (mL min^−1^)	N_2_ purity	Purging time (min)	Moles N_2_^b^ (nmol)	Moles NO_*x*_^b^ (nmol)	NH_3_ from NO_*x*_ (nmol s^−1^)
**100**	**99.999% (10 ppm NO**_***x***_**)**^**c**^	240	9.82 × 10^8^	9.82 × 10^3^	0.68
	**99.99% (100 ppm NO**_***x***_**)**			9.82 × 10^4^	6.8
	**99.9% (1000 ppm NO**_***x***_**)**			9.82 × 10^5^	68
**20**	**99.999% (10 ppm NO**_***x***_**)**	240	1.96 × 10^8^	1.96 × 10^3^	0.14
	**99.99% (100 ppm NO**_***x***_**)**			1.96 × 10^4^	1.4
	**99.9% (1000 ppm NO**_***x***_**)**			1.96 × 10^5^	14
**0**	**99.999% (10 ppm NO**_***x***_**)**	15 (prior to experiment at 20 mL min^-1^)	1.23 × 10^7^	1.23 × 10^2^	0.009
	**99.99% (100 ppm NO**_***x***_**)**			1.23 × 10^3^	0.09
	**99.9% (1000 ppm NO**_***x***_**)**			1.23 × 10^4^	0.9

^a^Arbitrarily assumed duration of a typical experiment.

^b^At 298 K and 1 atm.

^c^Assumes for illustration purposes that all of the impurities are NO_x_ compounds.

### Other sources of non-genuine NRR

Apart from NO_*x*_ compounds emerging from the sources discussed above, it is also important to briefly highlight other potential sources of the non-genuine ammonia formation in an NRR experiment. Similar to NO_*x*_, ammonia itself is a common atmospheric contaminant, although is often much easier to remove and control. More importantly, electrodes, electrocatalysts, electrolytes, solvents and any other component of the experimental setup that is nitrogen-based or was prepared using N-containing precursor(s) can be a source of contamination. In this scenario, ammonia can be either released spontaneously upon bringing different components into contact (*e.g*. immersing an electrode into a solution), or upon electrochemical stimuli. For example, materials like nitrides^24,25^ and phthalocyanines^26^ that have been tested as NRR catalysts were all found to decompose under reductive potentials with the formation of ammonia through a non-electrocatalytic reaction in aqueous electrolytes.

## Case studies

To further emphasise the importance of control over adventitious sources of nitrogen in an NRR experiment, we present several case studies based on published data for the most intensively studied aqueous electrolytes. We consider four significant families of catalysts—significant either because the published results are substantial, but are false-positives, or because of the attractiveness of the materials or mechanisms involved. Thus, the focus below is on metallic bismuth powder (hereinafter, Bi), carbon-supported gold nanoparticles (Au/C), nitrogen-containing carbon materials (CN_*x*_) and metal nitrides, all of which have been reported to exhibit reasonable electrocatalytic activity towards the NRR in aqueous electrolyte solutions, as listed in Supplementary Table 1.

**Fig. 2:**
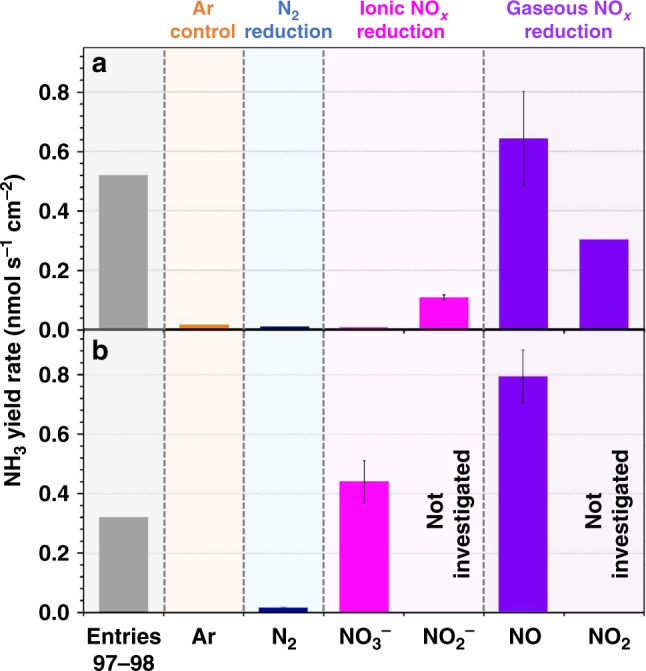
Bi and Au/C: NRR vs. NO_x_ reduction. Comparison of the NH_3_ formation rates for electrodes modified with **a** Bi (tested at −0.65 V vs. reversible hydrogen electrode, RHE) and **b** Au/C (tested at −0.30 V vs. RHE) reported in the literature (entries 97–98 in Supplementary Table 1) and measured in our laboratory in the presence of Ar, N_2_ and different NO_x_ compounds using 0.5 M K_2_SO_4_ aqueous electrolyte solutions (for further details, see ref. ^4^). All data are presented as an average ± standard deviation calculated for tests with three independent samples of each type.

We first consider the cases of Bi and Au/C with the initial key intention of highlighting the inability of other laboratories to reproduce the results originally reported in the literature (Fig. 2). The NRR rates for both types of materials were reported to be some of the highest in the field (entries 96–98 and 104 in Supplementary Table 1), while our experiments, undertaken with all potential sources of nitrogen-based contaminants strictly controlled and quantified, clearly demonstrate immeasurably low performance for both^3,4^.

Upon reaching this highly unfortunate outcome, we considered a range of possibilities that could result in false-positive NRR results by scrutinising the experimental procedures described in the relevant papers. The common feature of all these studies was the lack of any reasonable control, or quantification, of the NO_*x*_ contamination, notwithstanding the fact that bismuth and gold are known to efficiently catalyse the NO_*x*_ electroreduction to ammonia^27–30^. A range of tests in our laboratories with both ionic and gaseous NO_*x*_ compounds indeed confirmed that Bi and Au/C can produce NH_3_ quite efficiently with the rates of the order of 0.1-1.0 nmol s^−1^ cm^−2^ when these contaminants are present (Fig. 2). Of particular concern in this case was NO_(g)_ which can be reduced at the highest rates and at the least negative potentials^4^.

**Fig. 3:**
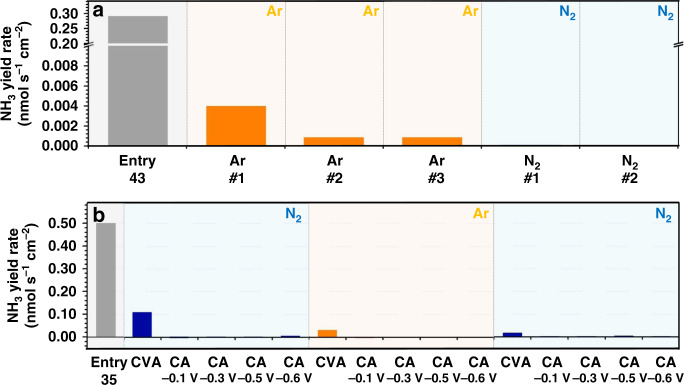
CN_x_ and VN: NRR vs. decomposition. NH_3_ formation during the electroreduction of **a** CN_x_- and **b** VN-modified electrodes previously reported in the literature (entries 35 and 43 in Supplementary Table 1), and as measured in our laboratory at different stages of a continuous test. Electrolyte solutions and potentials (vs. reversible hydrogen electrode) used in tests are provided in corresponding panels; further experimental details can be found in Supplementary Figs. 1 and 2 and ref. ^25^ (CVA = cyclic voltammetry, CA = chronoamperometry).

The other two examples considered herein belong to the family of nitrogen-containing NRR “catalysts”—N-doped carbon materials and transition metal nitrides. Even though the decomposition of such materials with the release of NH_3_ upon electroreduction has been clearly demonstrated in reports from us^25^ and others^24^, similar compounds are still being reported to exhibit reasonable activity. To reassess some of these results, we have synthesised CN_*x*_ by thermal decomposition of a zeolite imidazolate framework (see details in Supplementary Fig. 1) and VN via a high-temperature reaction between vanadium hydroxide precursor and NH_3_^25^, and tested their ability to catalyse the reduction of N_2_ with relevant aqueous electrolyte solutions (Fig. 3). In contrast to the corresponding literature reports, we did not use a new electrode for tests in Ar and N_2_, but undertook continuous tests with alternating atmosphere for both types of materials. For CN_*x*_, the data obtained in such manner clearly demonstrate that the freshly prepared material generates NH_3_ despite the absence of N_2_, most probably through the reductive decomposition of N-containing functionalities (Fig. 3a). This process eventually decelerates to the extent that the ammonia yield rate is below 0.002 nmol s^−1^ cm^−2^, and subsequent introduction of N_2_ to the system reveals unmeasurable catalytic activity of CN_*x*_ for the NRR. These results highlight the need for repeated reduction measurements in Ar, in order to confirm NH_3_ levels at background, before carrying out N_2_ based measurements on the same electrode.

The case of VN is conceptually similar, *i.e*. ammonia was produced irrespective of the gas present (Fig. 3b). The distinct feature, in this case, was that all of the NH_3_ was in fact formed during preliminary cyclic voltammetric tests, while subsequent reductive chronoamperometry decreased the concentration of ammonia in the solution, presumably, due to the adsorption on the negatively charged electrode surface. The latter was circumstantially confirmed by observation of a reasonable amount of NH_3_ being washed off the electrode surface after the tests (see ref. ^25^. for further details). We also note that no measures to remove and quantify adventitious NO_*x*_ were taken in the previous work on CN_*x*_ and metal nitrides (see Supplementary Table 1), indicating an additional potential source of ammonia in these studies.

The key conclusion we can draw based on the above case studies is that the materials reassessed herein do not exhibit any measurable catalytic activity for the NRR. At the same time, we are not in position to unambiguously claim that NO_*x*_ and/or material decomposition was the only source of NH_3_ in the revisited published work, but the evidence presented above suggests that this is likely to be the case. Thus, our aim is to provide alternative perspectives to those reported previously and allow readers to make their own judgement on the electrocatalytic activity of bismuth, gold, nitrogen-doped carbon, metal nitrides, and the wide variety of other materials listed in Supplementary Tables 1 and 2.

## Updated and simplified NRR protocol

The pernicious yet rarely recognised impact of NO_*x*_ on electrochemical NRR, as discussed and exemplified above, necessitates a provision of a separate summary of the established procedures for the removal and quantitative analysis of these contaminants, prior to the discussion of an updated NRR protocol, which appears later in this section.

### Analysis and elimination of NO_*x*_

First, it is necessary to quantify the concentrations of NO_*x*_ compounds in the N_2_ gas supply prior to undertaking any electrochemical or non-electrochemical control experiments. A key point is that gas purity as specified by the supplier cannot be taken as sufficient to validate any given cylinder of gas; variability can be the cause of false positives and much-wasted researcher time and resources. Gases such as N_2_O, NO, and NO_2_ can be directly detected and quantified using a gas chromatograph equipped with either electron capture detector (ECD; *~*2 nmol L^−1^ limit of detection for N_2_O^31,32^) or nitrogen chemiluminescence detector (NCD; *~*0.4 nmol L^−1^ and 0.3 nmol L^−1^ limits of detection for both NO and NO_2_, respectively^33^). Commercial NO_*x*_ analysers, commonly used for monitoring NO and NO_2_ levels in the atmosphere, also use NCD^34^, but are not equipped with the column for gas separation, leading to NO_*x*_ signal interferences by the presence of *e.g*. hydrocarbons in the sample. In addition, some NO_*x*_ analysers require a continuous sample flow at *~*0.7 L min^−1^, which may not be suitable for a laboratory setting.

An example of the gas chromatographic detection of the N_2_O impurity level in an N_2_ cylinder in our laboratory is provided in Supplementary Fig. 3 and Supplementary Table 3. The detected level is well below the manufacturer’s specifications, as one would hope. Nonetheless, the sensitivity shown in Table 1 of the typical experiments to possible variations in gas cylinder purity, necessitates that this gas analysis is carried out on a regular basis on every new cylinder of N_2_ to be used for the experiments.

NO and NO_2_ can also be analysed when the N_2_ gas is passed through reactive scrubbing solutions. Although this is a very basic experimental practice, it is important to note that the contact area and contact time between the bubbles and liquid should be maximised in such procedures. This is most easily achieved by including a packing material such as glass beads or pieces in the trap such that a long, tortuous path for the bubbles is created in a relatively tall trap. Further discussion of NO_*x*_-scrubbing system design parameters is provided in Supplementary Figs. 4 and 5.

Our recommendation is that KMnO_4_ solution is the reactive scrubber of choice in this context, being a powerful yet sufficiently stable oxidising agent and able to react rapidly with the dissolving NO_*x*_ species. The alkaline or acidic aqueous solutions often employed in the NRR studies are not as efficient as KMnO_4_. NO—likely the most problematic contaminant in the NRR context—is not very highly soluble in H_2_O (*ca* 1.9 mmol L^−1^ at 25 °C^35^), and might not be efficiently captured. In contrast, KMnO_4_ converts NO into the more soluble NO_2_^36^ that will rapidly transform into NO_2_^−^ and NO_3_^−^. As the reaction of N_2_O with KMnO_4_ is unproven we prefer to use the gas chromatographic approach to detection of N_2_O levels discussed above.

The scrubbing action is a function of bubble size, contact time, and concentration, *via* a combination of Henry’s and Fick’s laws, and hence it is absolutely necessary to demonstrate that the implemented NO_*x*_ scrubber provides sufficient purification under experimental conditions, including the flow rate to be used in the main experiments. Since strong oxidants can interfere with analysis, we recommend a second trap, containing a simple alkaline solution, be used in line with the main scrubber to allow reliable measurement of any remaining NO_*x*_ *via* the spectrophotometric Griess method^37^ or ion chromatography^38^. The NO_*x*_ concentration in this solution should remain at the initial background level (which should be in turn very close to the limit of detection) under all operating conditions. As explained below, analysis and reporting of this data before and *on completion* of the main NRR experiments should be a routine part of a rigorous protocol.

It is important to note that Henry’s law predicts relatively low saturated values of the N_2_O and NO compounds in the scrubber solutions and the experimental electrolyte. This in principle means that only very low values of reductive currents could be supported by these concentrations under static conditions. However, when the gas bubbles are directed over the electrode or used in a gas diffusion electrode, direct gas-to-surface adsorption is likely and Henry’s law is no longer the limiting factor. The need for further study of adsorption energies is discussed further below in Section 5.

Such a trap approach described above is not directly applicable to studies with non-aqueous electrolytes, which arguably provide the only known conditions that enable genuine NRR under low temperature and pressure^9^. The use of solid-state NO_*x*_ purifying columns^9^ is more convenient in this case, although if such columns are not available, several high-volume traps filled with an organic solvent of interest installed between the aqueous NO_*x*_ scrubber and the electrochemical cell might be used as well^39^.

Purifying ^15^N_2_ is somewhat more problematic given the desire to minimise the quantity of gas used and the need to also eliminate ^14^N_2_ from the system. For these reasons we recommend the scrubber described by Andersen et al. using an active metal reductant to remove the NO_*x*_ species^9^, followed by an alkaline trap that is (as above) used purely for analysis purposes to demonstrate no breakthrough of NO_*x*_ species.

Apart from entering the electrochemical cell together with N_2_, NO_*x*_ compounds spontaneously accumulate on most surfaces, as already mentioned above and well documented elsewhere^9,18–20^. Therefore, extensive purification of equipment needs to be implemented prior to all NRR experiments. Among other options, alkaline solutions present a good choice for the efficient removal of NO_*x*_. With the presence of alkali, the hydrolysed nitrogen oxide pieces, *viz*. HNO_2_ and HNO_3_, can be neutralised to NO_2_^−^ and NO_3_^−^, impeding the re-formation of gaseous NO_*x*_, which improves the cleaning effect^16^. Thus, to remove NO_*x*_ contaminations from the cell, electrodes and other labware, washing with alkaline solutions (pH ≥ 10 based on our experience) is an efficient method. Chemicals, *e.g*. electrolytes and catalytic materials contaminated with nitrogenous species can be purified through recrystallisation or annealing at an appropriate temperature^21^, while subsequent storage should be under vacuum or clean argon atmosphere. Contact time with ambient environment during handling and preparations should be as short as possible to minimise the unavoidable readsorption of ambient NH_3_ and NO_*x*_, which amount should be quantified through the Ar control experiments.

Most critically, we emphasise again that NO_*x*_ content in the gas pre-purification units, electrochemical cell, and any traps installed to capture the products of the reaction should be monitored alongside ammonia at all stages. This allows almost immediate identification of extraneous nitrogen compounds, that could be introduced either continuously with *e.g*. the N_2_ feed gas (through variations in supply leaks or entrainment), or through such routine operations like sampling during the experiment. NO_2_^−^/NO_3_^−^ determination by the Griess method^37^ is straightforward and the data should be shown alongside ammonia yield data on all histograms and graphs in all key experiments.

### Undertaking an NRR experiment

**Fig. 4:**
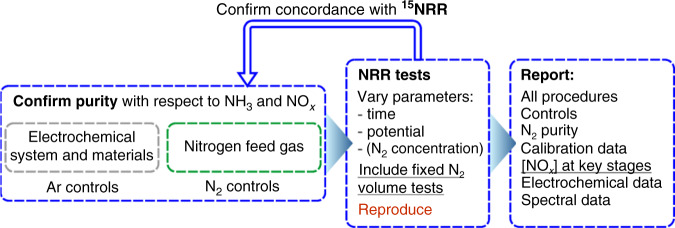
Updated and simplified NRR experimental protocol. Tests with a fixed and/or low volume of the N_2_ gas are recommended, as is the quantification of NO_x_ at every key step. Demonstration of the NRR as a function of time and potential is critical, while studies aiming at thorough kinetic analysis should also investigate the effect of N_2_ concentration (which can be either decreased or increased with respect to the experiments at 1 atm). More detailed explanation of each key stage has been reported previously by us^8,25^ and others^7,9^.

The protocol for undertaking a reliable NRR experiment described below and in Fig. 4 adopts all key features of the previously published recommendations^7–9^. Therefore, we provide only a brief summary of these critical aspects herein. Before commencing any tests, the purity of the gases to be employed (Ar, ^14^N_2_, and then ^15^N_2_) should be quantitatively determined, and improved if necessary, as discussed above. Tests should start with a set of controls to confirm the background levels of NH_3_ and NO_*x*_ of the experimental components, including scrubbers, gas lines, *all* components of the cell, sampling and analysis labware, etc. Then the assembled experimental system is tested, *with and without potential applied*, under pure argon atmosphere flowing at the rate to be used in the main NRR experiments to determine [NH_3_]^Ar^ and [NO_*x*_]^Ar^. Any increase in the amount of NH_3_/NO_*x*_ beyond the individual background levels at this stage should serve as a warning of the unsuitability of the employed conditions for reliable NRR experiments and should be addressed through the implementation of stricter purification procedures.

Further, experiments, at an open circuit, under exactly the same conditions but in the presence of ^14^N_2_ instead of Ar should be undertaken to identify the NH_3_/NO_*x*_ amounts in the system $[{\mathrm{NH}}_3]^{{\mathrm{N}}_2}$ and $[{\mathrm{NO}}_x]^{{\mathrm{N}}_2}$; any changes with respect to [NH_3_]^Ar^ and [NO_*x*_]^Ar^ indicate impurities in the N_2_ gas stream and should be dealt with by improved gas-scrubbing. In these controls, we recommend that additional efforts are made to remove and analyse any NH_3_/NO_*x*_ that can be adsorbed on the working electrode through rigorous washing, especially if a high-surface area substrate is used. If NO_*x*_ level remains unchanged and identical under Ar and ^14^N_2_ (ideally near the limit of detection) then the main NRR experiments can begin, determining $[{\mathrm{NH}}_3]_{{\mathrm{NRR}}}^{{\mathrm{N}}_2}$ and $[{\mathrm{NO}}_x]_{{\mathrm{NRR}}}^{{\mathrm{N}}_2}$ at various potentials. If the amount of NH_3_ reproducibly demonstrates a significant increase (see discussions of the NRR reliability **Criterion I** above) while the amount of NO_*x*_ does not change beyond the background $[{\mathrm{NO}}_x]^{{\mathrm{N}}_2}$ level, experiments with ^15^N_2_ following the same procedure should be undertaken. The same approach to NO_*x*_ compound removal and quantification needs to be applied. The amount of ^15^NH_3_ and ^14^NH_3_ in ^15^NRR experiments should be quantified using reliable procedures^10,20^. As ^14^N_2_ can be difficult to completely remove from these experiments it is to be expected that both ^15^NH_3_ and ^14^NH_3_ can be present and both should be quantified with their relevant calibration curves. If the total NH_3_ in this experiment is similar, within an experimental error, to the amount of ^14^NH_3_ obtained in the main ^14^N_2_ experiments, successful achievement of the NRR can be claimed.

We recommend that the full protocol described here be applied at the outset of testing of a new catalyst/electrode/electrolyte to detect any spurious nitrogenous sources. Once they have been established as being not significant, then the NO_*x*_ analysis may not be necessary in routine experiments. However, we recommend that authors, reviewers and editors should always expect to see headline results being quoted alongside full protocol NO_*x*_ data for the same experiment.

Where a high yielding NRR process has been robustly proven previously to be genuine, it can be reasonable to refer back to the earlier work for proof. The only such process in Supplementary Tables 1 and 2 is the Li-mediated system. Nonetheless, it is important to clearly justify the basis of the reliance on the previous data; even small variations, for example in materials used, can introduce contaminants that could produce false-positive effects and improvements.

A key distinctive feature proposed herein, and not explicitly emphasised in previous publications, is the recommendation to minimise any possible NO_*x*_ contamination introduced in the system via minimising the amount of gas used in the experiments. This can be easily achieved by using a fixed, relatively small volume of N_2_ for the NRR tests, as opposed to the commonly undertaken “flowing gas” experiments. This is shown in the protocol (Fig. 4) as an important qualification test for promising NRR experiments, before proceeding to the more expensive ^15^N_2_ experiments.

To emphasise this point, the maximal possible rates of the ammonia formation through the NO_*x*_ reduction reaction are compared for these two types of experiment in Table 1. For the standard flowing scenario, any NO_*x*_ contained in the feeding gas will be continuously supplied to the electrochemical cell and serve as a continuous source of electrosynthesised ammonia. The danger here is that these NO_*x*_ compounds might be very hard to detect in an electrochemical experiment, as their reduction will be rapid which will prevent a build-up of detectable concentrations. In contrast, when the amount of N_2_, and consequently any accompanying NO_*x*_ contaminations is fixed and limited, the latter will be eventually depleted and will not contribute to the electrosynthesis of ammonia.

If the efficient operation of an NRR system requires intensified mass transport, this can be provided either through stirring the electrolyte solutions or using a rotating disk electrode. If the actual gas flow is deemed necessary, recirculation of a fixed volume of gas can be implemented^9^, although one should be cautious of additional sources of contamination that might arise from the gas pump. Regardless, a fixed volume experiment is a strong evidence of genuine NRR and, though the yield rate may be lower because of the modified mass transport rates, we recommend such experiments in the experimental protocol to quickly identify impurity problems that stem from large volumes of flowing gas.

Finally, based on the analysis of the recent publications, not only in the NRR but in the broad electrocatalysis field, we feel it is necessary to emphasise an obvious, but ubiquitously overlooked requirement to reproduce the experimental results and report data in a manner that provides a quantitative measure of the reliability of measurements, *e.g*. as mean ± one standard deviation calculated from repeated experiments. The latter should be based on measurements with at least three *independently prepared samples*, not just several repeats of measurements with the same sample only. This approach should be always applied to the best-performing catalyst, as well as to all experiments that underpin the key claims of a study.

Notwithstanding a specific focus of this perspective on the electrochemical reduction of N_2_, we note that many of the control experiments, purification and analytical procedures can and should be applied to other modes of the conversion of dinitrogen to ammonia that struggle to achieve practically relevant yield rates. In the first place, this is pertinent to the photochemical N_2_ fixation studies, many of which produce even less ammonia than the NRR.

## Understanding NRR through computational chemistry

A common feature of the recent NRR literature is the use of density functional theory (DFT) calculations to complement and support the experimental work, which is, in principle, laudable and can be expected to accelerate the development of this important field. Indeed, the depth of our understanding of the catalytic, and more specifically electrocatalytic, phenomena has increased massively with the rise of the computational chemistry. The most commonly applied mode of such theoretical analysis in the context of heterogeneous electrocatalysis involves the calculation of thermodynamic energy profiles for the hypothesised reaction pathways involving several elementary steps including adsorption/desorption, charge transfer and chemical transformations of the adsorbed species. Data calculated in this manner can be either used to (i) rationalise the experimental observations, *i.e*. explain why a particular catalyst is active/inactive for the process of interest, or (ii) predict theoretically the electrocatalytic properties of a range of materials to guide future experimental studies.

To non-specialists in computational chemistry, like the authors of the present paper, either of these outcomes is typically the key aim of involving computational chemistry in the electrocatalytic studies, including those focusing on the NRR. Many recent papers on dinitrogen electroreduction include a DFT section commonly demonstrating a (partially) thermodynamically feasible profile of the N_2_ conversion to NH_3_ over a flagship catalyst at a given potential to draw a conclusion of the kind “theory confirms experiment”. More detailed, and often more useful, analysis aims to establish the key features of a given material that underpin the expected high catalytic performance, by comparisons to a range of relevant models. However, this theoretical analysis is sometimes subject to conceptual problems that are obvious even to a non-specialist in DFT. In particular, some papers, including those published in high-profile journals, discuss the feasibility of charge-transfer steps only, the thermodynamics of which can be favourably adjusted by “applying” an increasingly (sometimes unrealistic) negative potential, but ignore the very significant energy barriers, of the order of a few eV, for other steps such as N_2_ adsorption or NH_3_ desorption, which do not depend on the potential. For example, theoretical data of this kind appear in publications 3, 23, 29, 40, 41, 51, 55, 82, 92, 97, 105, 109, 112, 116, 122, and 123 in Supplementary Table 1. Significant disagreement between the theoretically predicted potentials required for the NRR with those reported experimentally is also not uncommon. It seems that DFT studies are sometimes included because it is *de rigueur* (fashionable) to do so, not because it actually supports or explains the experimental results.

The most insightful theoretical studies in the NRR context, from our perspective, are those that build upon the theory in the first place. This can be either an overarching analysis of the general trends, plausible mechanisms and key limiting steps for the NRR catalysed by a broad family of materials^2,40–43^, or a more specific investigation of one particular highly promising system^44–48^. Ideally, this kind of theoretical work should be followed by experiments to confirm the predicted activity, though unfortunately, we are not aware of any NRR work that has successfully followed that pathway. On the contrary, the lack of concordance of theory to experiments for some materials, *e.g*. transition metal nitrides, has been reported by us^25^ and others^24^.

The specific aspect of the NRR that is not considered in the overwhelming majority of the theoretical studies is the competition between the adsorption on the electrocatalyst surface of nitrogen and hydrogen. This important problem has been highlighted by Nørskov and co-workers in their seminal paper published in 2012^2^, but surprisingly is very rarely investigated in more recent theoretical work. Given that adsorption of N_2_ on the majority of surfaces traditionally considered in the electrocatalytic context is typically much weaker than that of H, O, NH_3_ or even the notorious NO_*x*_, these competing reactions need to be allowed for. Analysing the catalytic activity towards the NRR in the context of these competing processes is like trying to light a match in the wind. Comparisons of the adsorption energies for all species present in the system under relevant electrocatalytic conditions should be undertaken, and only those materials that demonstrate preference to the adsorption of N_2_ should be considered as promising NRR catalysts. Arguably, this analysis should be the second step of the theoretical work on the NRR, the first being the confirmation of the thermodynamic stability of the selected material itself, under relevant conditions. Future work should also involve deeper analysis of the kinetic aspects of each elementary step, *i.e*. calculations of the activation energies, in accordance with the recent developments in the field of computational chemistry^49–53^.

## Future directions and perspectives

The concept of the direct electrochemical reduction of dinitrogen to ammonia, a compound of immense current and potentially even higher future technological significance, captivates scientists, and stimulates a highly active research. The field has intensified exponentially over the recent years, but the analysis of the key reports published to date indicates that the broadly adopted experimental procedures for the N_2_ electroreduction require critical reassessment and improvements.

The first key aim of this perspective is to serve as a cautionary guide to researchers interested in the NRR, in particular through the demonstration of the irreproducibility and potential unreliability of many previous publications. In our view, in accord with the authors of ref. ^9^, the possibility of the direct N_2_ reduction to ammonia with aqueous electrolytes remains unproven, notwithstanding the publication of more than a hundred papers reporting on “successful” aqueous NRR with a multitude of electrode materials. Many of these papers specifically note that the published protocol procedures^7–9^ were strictly followed, though in reality this was commonly not the case with key controls and experimental aspects being ignored, as elaborated above. Herein, we specifically emphasise the overlooked problem of the NO_*x*_ contaminants, which are likely to be the true origin of the majority of the recent “successful” NRR experiments. This forms the basis of the second purpose of the present paper: to provide a simplified experimental guide, combining all key aspects published before^7–9^ with updated recommendations for minimising the possibility of N-based contaminants contributing substantially to the observations. Recognising this issue early in an experimental program can only serve to save time, resources and careers.

Our third objective herein was to formulate reliability and performance targets that might assist the evolution of the NRR field into an area of research that has strong credibility from the perspective of funding bodies and investors. We encourage researchers to aim for three “greens” in the criteria described above, *viz*. strive for practical NH_3_ yield rates, fully reliable and quantitative experiments and complete elimination of the reduction of the oxidised forms of nitrogen as a source of ammonia. Although the ammonia yield rate of 10 nmol s^−1^ cm^−2^ as the minimal target for the practically relevant process might appear challenging at this stage, we note that it is still at least an order of magnitude lower than the industrially relevant targets^5^. In no respect do we aim to discourage fundamental research on the NRR, where such high productivity is not being achieved, but rather we aim to help distinguish between the processes that currently pertain to the purely fundamental domain (but still should prove their reliability by scoring “orange” in the NH_3_ yield rate criterion) and those that can potentially support the development of the *Ammonia Economy* in the foreseeable future^1^. Notably those that cannot score green in this criterion can hardly claim to be presenting a practical breakthrough in the field, as unfortunately is currently so frequently the case in the literature. At the same time, such research can be exceptionally useful to the field in a different way, *viz*. through the studies of the reaction kinetics and mechanism(s), identification of the true active state and surface sites of the catalyst, key intermediates and rate-limiting stages of the N_2_ reduction. It is undisputable that genuine scientific innovation can be only underpinned by robust fundamental knowledge, and the NRR is of course no exception. Importantly, fundamental research will not only aid the development of the ammonia electrosynthesis technologies, but is also likely to contribute to the development of the science of electrocatalysis, as has happened before in investigations of the hydrogen and oxygen electrode reactions.

The recent story of “false-success” of the NRR can be seen as a quintessence of the experimental and theoretical flaws, misconceptions and even ignorance that have recently spread over the broad contemporary field of *electrochemical materials*. Lack of attention to fundamental principles, lack of any effort to prove the reliability of the reported results, propagation of mistakes from one paper to many others—all of these and other problems incompatible with robust scientific research are currently seeing a “renaissance” through these NRR studies. The electrosynthesis of ammonia from dinitrogen presents a comparatively new and an intensely topical challenge in contemporary science, and hence can provide an appealing pathway towards scientific success. Unfortunately, such “success” is often achieved through the rushed publication of results that are irreproducible and unreliable.

Herein, we call the electrocatalysis community to rehabilitate the reputation of the NRR field. Apart from the implementation of improved instrumental practices, we also encourage scientists to intensify the exchange of materials and experience between independent laboratories, which will minimise the risk of mistakes. With broad support, this might evolve into the establishment of electrocatalyst “certification laboratories”, as has been already implemented in other important fields. Our own laboratories are open to visitors to bring, or send, samples for testing, an offer frequently made yet seldom taken up. Such cross-verification of results will provide the transparency in experimental protocols that is urgently required to render studies of the nitrogen reduction reaction a domain of reliable research.

## Supplementary information

Supplementary Information
