# Supplementary material for: Identification and elimination of false positives in electrochemical nitrogen reduction studies
Source: Nat Commun. 2020 Nov 3;11:5546. doi: 10.1038/s41467-020-19130-z (PMC7641139; doi:10.1038/s41467-020-19130-z)
Supplement: Supplementary file 1 — Supplementary Information [file 41467_2020_19130_MOESM1_ESM.pdf]

SUPPLEMENTARY INFORMATION FOR:

Identification and elimination of false positives in electrochemical nitrogen reduction studies

Jaecheol Choi[1,2], Bryan H. R. Suryanto[1], Dabin Wang[1], Hoang-Long Du[1,2], Rebecca Y. Hodgetts[1,2], Federico M. Ferrero Vallana[1], Douglas. R. MacFarlane[1,2] and Alexandr N. Simonov[1,2]

[1]School of Chemistry and the [2]ARC Centre of Excellence for Electromaterials Science, Monash University, Victoria, Australia

Correspondence and requests for materials should be addressed to:
D.R.M. (email: douglas.macfarlane@monash.edu), A.N.S. (email: alexandr.simonov@monash.edu)

TABLE OF CONTENTS

		**Page**
**Supplementary Table 1.**	Summary of aqueous NRR results reported between 2010 and 2020.	3
**Supplementary Table 2.**	Summary of non-aqueous NRR results reported between 1993 and 2020.	10
**Supplementary Figure 1.**	Synthesis, characterisation and testing of CN_x materials.	11
**Supplementary Figure 2.**	Ammonia generation by a vanadium nitride electrode.	12
**Supplementary Figure 3.**	Gas chromatographic analysis of the N_2O content in $^{14}N_2$, $^{15}N_2$ and Ar gas supplies.	13
**Supplementary Table 3.**	Potential NH_3 formation rates from N_2O in flowing-gas and static NRR experiments.	14
**Supplementary Figure 4.**	Experimental setup for NO_x scrubbing.	15
**Supplementary Figure 5.**	Experimental demonstration of NO_x scrubbing.	16
**Supplementary References**		17

Supplementary Table 1. Summary of aqueous NRR results reported between 2010 and 2020.

No.	Year	Material	N-based material	Electrolyte	*E* (V vs. RHE)	FE (%)	Yield rate (nmol s^{-1} cm^{-2})	Quantitative $^{15}N_2$ test	NO_x control	Ref.
**1**	2010	**polypyrrole film**	Y	0.1 M Li_2SO_4	-0.165	--	**0.018**	**N**	**N**	1
**2**	2016	**Au/TiO_2**	N	0.1 M HCl	-0.2	8.1	**0.35**	**N**	**N**	2
**3**	2016	**Au**	Y	0.1 M KOH	-0.2	4.0	**0.027**	**N**	**N**	3
**4**	2016	**Au-CeO_x/RGO**	Y	0.1 M HCl	-0.2	10	**0.14**	**N**	**N**	4
**5**	2017	**γ-Fe_2O_3**	N	0.1 M KOH	-0.4	2.0	**0.016**	**N**	**N**	5
**6**	2017	**Fe_2O_3-CNT**	Y	0.5 M KOH	-2 vs AgCl	0.30	**0.019**	**N**	**N**	6
**7**	2017	**Fe_2O_3-CNT**	Y	$KHCO_3$	-2 vs AgCl	0.20	**0.004**	**N**	**N**	7
**8**	2017	**polyimide/C**	Y	0.5 M Li_2SO_4	-0.4	2.9	**0.008**	**N**	**N**	8
**9**	2017	**Mo**	Y	0.01 M H_2SO_4	-0.4	0.70	**0.031**	**N**	**N**	9
**10**	2017	**FeMOF**	Y	2 M KOH	1.2	1.4	**2.1**	**N**	**N**	10
**11**	2018	**Ru**	N	0.01 M HCl	-0.1	1.0	**0.010**	**N**	**N**	11
**12**	2018	**Fe_3O_4**	N	0.1 M Na_2SO_4	-0.4	2.6	**0.056**	**N**	**N**	12
**13**	2018	**RuPt**	N	1 M KOH	0.123	13	**0.30**	**N**	**N**	13
**14**	2018	**$Pd_{0.2}Cu_{0.8}$/rGO**	N	0.1 M KOH	-0.2	0.70	**0.046**	**N**	**N**	14
**15**	2018	**Pd**	N	0.1M PBS	0.1	8.2	**0.022**	**N**	**N**	15
**16**	2018	**Cr_2O_3**	Y	0.1 M HCl	-0.7	8.6	**0.092**	**N**	**N**	16
**17**	2018	**Ru/CN_x**	Y	0.05 M H_2SO_4	-0.2	30	**0.50**	**N**	**N**	17
**18**	2018	**Nb_2O_5**	Y	0.1 M HCl	-0.55	9.3	**0.071**	**N**	**N**	18

No.	Year	Material	N-based material	Electrolyte	*E* (V vs. RHE)	FE (%)	Yield rate (nmol s^{-1} cm^{-2})	Quantitative $^{15}N_2$ test	NO_x control	Ref.
**19**	2018	**Fe/Fe_3O_4**	N	0.1 M PBS	-0.3	8.3	**0.003**	**N**	**N**	19
**20**	2018	**Ag**	N	0.1 M HCl	-0.6	4.8	**0.046**	**N**	**N**	20
**21**	2018	**C**	N	0.1 M Na_2SO_4	-0.3	6.9	**0.26**	**N**	**N**	21
**22**	2018	**NbO_2**	N	0.05 M H_2SO_4	-0.65	20	**0.19**	**N**	**N**	22
**23**	2018	**Y_2O_3**	N	0.1 M Na_2SO_4	-0.9	2.5	**0.11**	**N**	**N**	23
**24**	2018	**d-MoS_2**	Y	0.1 M Na_2SO_4	-0.4	8.3	**0.19**	**N**	**N**	24
**25**	2018	**Au/CN_x**	Y	0.1 M HCl	-0.2	12	**0.038**	**N**	**N**	25
**26**	2018	**Au/C_3N_4**	Y	0.005 M H_2SO_4	-0.1	11	**0.031**	**N**	**N**	26
**27**	2018	**Fe/N-CNT**	Y	0.1 M KOH	-0.2	9.3	**0.29**	**N**	**N**	27
**28**	2018	**B-TiO_2**	N	0.1 M Na_2SO_4	-0.8	3.4	**0.024**	**N**	**N**	28
**29**	2018	**B_4C**	N	0.1 M HCl	-0.75	16	**0.043**	**N**	**N**	29
**30**	2018	**Au**	N	0.1 M HCl	-0.2	6.1	**0.25**	**N**	**N**	30
**31**	2018	**Mn_3O_4**	N	0.1 M Na_2SO_4	-0.8	3.0	**0.038**	**N**	**N**	31
**32**	2018	**CN_x**	N	0.1 M HCl	-0.2	1.5	**1.0**	**N**	**N**	32
**33**	2018	**SnO_2**	N	0.1 M Na_2SO_4	-0.8	1.2	**0.15**	**N**	**N**	33
**34**	2018	**TiO_2**	N	0.1 M Na_2SO_4	-0.7	2.5	**0.092**	**N**	**N**	34
**35**	2018	**VN**	N	0.05 M H_2SO_4	-0.2	6.5	**0.50**	**N**	**N**	35
**36**	2018	**TiO_2-rGO**	N	0.1 M Na_2SO_4	-0.9	3.3	**0.099**	**N**	**N**	36
**37**	2018	**Cr_2O_3**	N	0.1 M Na_2SO_4	-0.9	6.8	**0.050**	**N**	**N**	37

No.	Year	Material	N-based material	Electrolyte	*E* (V vs. RHE)	FE (%)	Yield rate (nmol s^{-1} cm^{-2})	Quantitative $^{15}N_2$ test	NO_x control	Ref.
**38**	2018	**FeOOH**	N	0.5 M $LiClO_4$	-0.75	6.7	**0.076**	**N**	**N**	38
**39**	2018	**a-Fe_2O_3**	N	0.1 M KOH	-0.9 vs AgCl	8.3	**0.024**	**N**	**N**	39
**40**	2018	**MoO_3**	N	0.1 M HCl	-0.5	1.9	**0.48**	**N**	**N**	40
**41**	2018	**B-graphene**	N	0.05 M H_2SO_4	-0.5	11	**0.16**	**N**	**N**	41
**42**	2018	**Rh**	Y	0.1 M KOH	-0.2	0.20	**0.13**	**N**	**N**	42
**43**	2018	**CN_x**	Y	0.05 M H_2SO_4	-0.9	1.4	**0.29**	**N**	**N**	43
**44**	2018	**$Bi_4V_2O_{11}/CeO_2$**	Y	0.1 M HCl	-0.2	10	**0.76**	**N**	**N**	44
**45**	2018	**CN_x**	Y	0.1 M KOH	-0.3	10	**0.94**	**N**	**N**	45
**46**	2018	**Mo_2C/C**	N	0.5 M Li_2SO_4	-0.3	7.8	**0.18 nmol s^{-1} mg^{-1}**	**N**	**N**	46
**47**	2018	**Au**	Y	0.5 M $LiClO_4$	-0.5	15	**0.064**	**N**	**N**	47
**48**	2018	**CN_x**	Y	0.25 M $LiClO_4$	-1.2	11	**0.16**	**N**	**N**	48
**49**	2018	**MoS_2**	Y	0.1 M Na_2SO_4	-0.5	1.2	**0.081**	**N**	**N**	49
**50**	2018	**Au/CN_x**	Y	0.1 M HCl	-0.1	22	**0.59**	**N**	**N**	50
**51**	2018	**C_3N_4**	Y	0.1 M HCl	-0.2	12	**0.26**	**N**	**N**	51
**52**	2018	**Mo_2N**	Y	0.1 M HCl	-0.3	4.5	**1.2 nmol s^{-1} mg^{-1}**	**N**	**N**	52
**53**	2019	**$LiMn_2O_4$**	Y	0.1 M HCl	-0.5	7.4	**0.052**	**N**	**N**	53
**54**	2019	**CoS_x/CN_x**	Y	0.05 M H_2SO_4	-0.2	6.5	**0.082**	**N**	**N**	54
**55**	2019	**TiO_2**	N	0.1 M HCl	-0.12	6.5	**0.059**	**N**	**N**	55
**56**	2019	**CoO**	N	0.1 M Na_2SO_4	-0.6	8.3	**0.096**	**Y**	**N**	56

No.	Year	Material	N-based material	Electrolyte	*E* (V vs. RHE)	FE (%)	Yield rate (nmol s^{-1} cm^{-2})	Quantitative $^{15}N_2$ test	NO_x control	Ref.
**57**	2019	**NiO/graphene**	N	0.1 M Na_2SO_4	-0.7	7.8	**0.061**	**N**	**N**	57
**58**	2019	**Fe_3C/CN_x**	Y	0.1 M KOH	-0.4	2.7	**0.10**	**N**	**N**	58
**59**	2019	**B**	N	0.1 M HCl	-0.14	4.8	**0.051**	**N**	**N**	59
**60**	2019	**Ta_2O_5**	Y	0.1 M HCl	-0.7	8.9	**0.26**	**N**	**N**	60
**61**	2019	**Mo/CN_x**	Y	0.1 M KOH	-0.3	15	**0.56**	**Y**	**N**	61
**62**	2019	**FePc**	Y	0.1 M Na_2SO_4	-0.3	111	**2.3**	**N**	**N**	62
**63**	2019	**W_2N_3**	Y	0.1 M KOH	-0.2	12	**0.038**	**N**	**N**	63
**64**	2019	**TiN**	Y	0.1 M Na_2SO_4	-0.6	9.1	**0.33**	**Y**	**N**	64
**65**	2019	**PdCuIr**	N	0.1 M Na_2SO_4	-0.3	5.3	**0.088**	**N**	**N**	65
**66**	2019	**$C\text{-}Ti_xO_y/C$**	N	0.1 M $LiClO_4$	-0.4	18	**0.15**	**Y**	**N**	66
**67**	2019	**Bi**	Y	0.1 M Na_2SO_4	-0.8	11	**0.042**	**N**	**N**	67
**68**	2019	**MoS_2/rGO**	Y	0.1 M $LiClO_4$	-0.45	4.6	**0.041**	**N**	**N**	68
**69**	2019	**$\gamma\text{-}Fe_2O_3/CN_x$**	Y	0.1 M HCl	-0.1	12	**1.2**	**N**	**N**	69
**70**	2019	**B-COF**	Y	0.1 M KOH	-0.2	45	**0.21**	**Y**	**Y**	70
**71**	2019	**S-rich MoS_2**	Y	0.1 M Li_2SO_4	-0.2	9.8	**7.1**	**Y**	**N**	71
**72**	2019	**$Ti_3C_2T_x$**	N	0.5 M Li_2SO_4	-0.1	4.6	**0.077**	**N**	**N**	72
**73**	2019	**Fe_2O_3/TiO_2**	Y	1 M KOH	-0.577	0.30	**2.7**	**N**	**N**	73
**74**	2019	**Pd_3Cu_1**	Y	1 M KOH	-0.25	1.2	**0.13**	**N**	**N**	74
**75**	2019	**Mo_2C**	Y	0.1 M HCl	-0.3	8.1	**0.62**	**N**	**N**	75

No.	Year	Material	N-based material	Electrolyte	E (V vs. RHE)	FE (%)	Yield rate (nmol s^{-1} cm^{-2})	Quantitative $^{15}N_2$ test	NO_x control	Ref.
**76**	2019	**CN_xP_y**	Y	0.1 M HCl	-0.2	4.2	**0.13**	**N**	**N**	76
**77**	2019	**Ru/CN_x**	Y	0.1 M HCl	-0.21	7.5	**0.060**	**N**	**N**	77
**78**	2019	**CuO/rGO**	N	0.1 M Na_2SO_4	-0.75	3.9	**0.18**	**N**	**N**	78
**79**	2019	**PdRu**	N	0.1 M HCl	-0.2	2.4	**0.36**	**N**	**N**	79
**80**	2019	**Au**	N	0.1 M Na_2SO_4	-0.2	13	**0.15**	**N**	**N**	80
**81**	2019	**Fe/CN_x**	Y	0.1 M KOH	0	57	**0.12**	**Y**	**N**	81
**82**	2019	**Bi**	Y	0.2 M Na_2SO_4	-0.6	12	**0.045**	**Y**	**N**	82
**83**	2019	**MnO**	N	0.1 M Na_2SO_4	-0.39	8	**0.11**	**N**	**N**	83
**84**	2019	**$K_2Ti_4O_9$**	N	0.1 M KOH	-0.5	5.9	**0.037**	**N**	**N**	84
**85**	2019	**Cr_2O_3/rGO**	N	0.1 M HCl	-0.7	3.5	**0.054**	**N**	**N**	85
**86**	2019	**La_2O_3**	N	0.1 M Na_2SO_4	-0.8	4.8	**0.022**	**N**	**N**	86
**87**	2019	**Co/CN_x**	Y	0.1 M Na_2SO_4	-0.7	22	**0.16**	**N**	**N**	87
**88**	2019	**Ag_3Cu**	Y	0.1 M Na_2SO_4	-0.5	13	**0.16**	**N**	**N**	88
**89**	2019	**MoO_2**	N	0.1 M HCl	-0.15	8.2	**0.20**	**N**	**N**	89
**90**	2019	**Black P**	N	0.01 M HCl	-0.7	3.1	**0.10**	**Y**	**N**	90
**91**	2019	**B**	N	0.1 M Na_2SO_4	-0.8	4.0	**0.022**	**N**	**N**	91
**92**	2019	**BN**	Y	0.01 M HCl	-0.75	4.7	**0.037**	**N**	**N**	92
**93**	2019	**CN_x**	Y	0.005 M H_2SO_4	-0.4	10	**0.36**	**N**	**N**	93
**94**	2019	**F-Graphene**	N	0.1 M Na_2SO_4	-0.7	4.2	**0.030**	**N**	**N**	94

No.	Year	Material	N-based material	Electrolyte	*E* (V vs. RHE)	FE (%)	Yield rate (nmol s^{-1} cm^{-2})	Quantitative $^{15}N_2$ test	NO_x control	Ref.
**95**	2019	**Au/CoO_x**	N	0.05 M H_2SO_4	-0.5	19	**0.25**	**N**	**N**	95
**96**	2019	**AuNi**	Y	0.05 M H_2SO_4	-0.14	68	**0.24**	**N**	**N**	96
**97**	2019	**Bi**	Y	0.5 M K_2SO_4	-0.6	66	**14**	**Y**	**N**	97
**98**	2019	**Au/C**	N	0.5 M K_2SO_4	-0.3	23	**0.33**	**N**	**N**	97
**99**	2019	**Pt/C**	N	0.5 M K_2SO_4	-0.025	1.0	**0.033**	**N**	**N**	97
**100**	2019	**CoS/TiO_2**	Y	0.1 M Na_2SO_4	-0.55	29	**0.81**	**N**	**Y**	98
**101**	2019	**N-NiO/C**	Y	0.1 M $LiClO_4$	-0.5	7.3	**0.19**	**Y**	**N**	99
**102**	2019	**Fe_3Mo_3C/C**	N	1 M KOH	-0.5	0.30	**0.21**	**N**	**N**	100
**103**	2019	**SnS_2/Ni**	Y	0.1 M Na_2SO_4	-0.5	11	**0.92**	**N**	**N**	101
**104**	2019	**Au/Boron organic polymer**	Y	0.1 M HCl	-0.2	10	**0.74**	**N**	**N**	102
**105**	2019	**Cu/CN_x**	Y	0.1 M KOH	-0.35	14	**0.87**	**N**	**N**	103
**106**	2019	**Pd_3Pb**	Y	0.1 M Na_2SO_4	-0.2	22	**0.060**	**N**	**N**	104
**107**	2019	**Cl-graphdiyne**	N	0.1 M HCl	-0.45	8.7	**0.18**	**N**	**N**	105
**108**	2019	**$rGO/Fe/Fe_3O_4$**	Y	0.2 M $NaHCO_3$	-0.3	6.3	**0.13**	**N**	**N**	106
**109**	2019	**$CoFe_2O_4$**	N	0.1 M Na_2SO_4	-0.4	6.2	**0.042**	**N**	**N**	107
**110**	2019	**Cu/Polyimide**	Y	0.1 M KOH	-0.3	6.6	**0.20**	**N**	**N**	108
**111**	2019	**O-CNT**	Y	0.1 M HCl	-0.6	5.0	**0.033**	**N**	**N**	109
**112**	2019	**PdCu**	Y	0.5 M LiCl	-0.1	12	**0.58**	**N**	**Y**	110
**113**	2019	**Co-doped $FePS_3$**	N	0.1 M KOH	-0.4	3.4	**0.24**	**N**	**N**	111

No.	Year	Material	N-based material	Electrolyte	*E* (V vs. RHE)	FE (%)	Yield rate (nmol s^{-1} cm^{-2})	Quantitative $^{15}N_2$ test	NO_x control	Ref.
**114**	2019	**Au**	Y	0.1 M Li_2SO_4	-0.3	73	**0.15**	**N**	**N**	112
**115**	2019	**CN_x/NiO /Graphite**	Y	0.1 M HCl	-0.2	30	**0.12**	**N**	**N**	113
**116**	2019	**Fe_3C/C**	Y	0.05 M H_2SO_4	-0.2	9.2	**0.21**	**N**	**N**	114
**117**	2019	**Fe-doped TiO_2**	N	0.5 M $LiClO_4$	-0.4	26	**0.042**	**N**	**N**	115
**118**	2019	**P/SnO_{2-x}**	N	0.1 M Na_2SO_4	-0.4	15	**0.080**	**Y**	**Y**	116
**119**	2019	**$RuMoS_2$**	N	0.01 M HCl	-0.2	18	**0.067**	**Y**	**N**	117
**120**	2020	**$NbSe_2$**	Y	0.1 M Na_2SO_4	-0.45	7.0	**1.5**	**Y**	**N**	118
**121**	2020	**Au/Fe_3O_4**	Y	0.1 M KOH	-0.2	11	**0.18**	**Y**	**N**	119
**122**	2020	**$IrTe_4$**	Y	0.1 M KOH	-0.2	15	**0.83**	**Y**	**N**	120
**123**	2020	**CN_xB_y**	Y	0.1 M HCl	-0.2	11	**0.27**	**N**	**N**	121
**124**	2020	**Mo/FeS_2**	Y	0.1 M KOH	-0.2	14	**0.41 nmol s^{-1} mg^{-1}**	**Y**	**N**	122
**125**	2020	**$W_{18}O_4$/Fe**	N	0.25 M $LiClO_4$	-0.15	20	**0.40 nmol s^{-1} mg^{-1}**	**N**	**N**	123
**126**	2020	**CN_x**	Y	0.1 M HCl	-0.1	62	**0.041**	**Y**	**N**	124
**127**	2020	**Fe/SnO_2**	Y	0.1 M HCl	-0.3	20	**14**	**Y**	**N**	125

Colour codes:

Green: High yield rate ≥ 10 nmol s^{-1} cm^{-2}; key experiments executed according to protocols with clearly outlined experimental details.

Orange: Moderate yield rate between 0.1-10 nmol s^{-1} cm^{-2}; partial provision of the required key experiments, *e.g.* incomplete quantification of NO_x, ^{15}NRR experiment under single set of conditions only with no time dependence.

Red: Low yield rate < 0.1 nmol s^{-1} cm^{-2}; key experiments and/or experimental details missing; fundamental flaws in the experimental procedures, *e.g.* identifiable H/D exchange in the analysis of $^{15}NH_3$ by ^{1}H-NMR.

Supplementary Table 2. Summary of non-aqueous NRR results reported between 1987 and 2020.

No.	Year	Material	N-based material	Electrolyte	*E* (V)	FE (%)	Yield rate (nmol s^{-1} cm^{-2})	Quantitative $^{15}N_2$ test	NO_x control	Ref.
**1**	1987	**0.05 M Cp_2TiCl_2**	N	0.25 M $LiClO_4$ in MeOH + 0.25 M catechol/$Mg(OAc)_2$	-2.2 vs. Ag wire	2	**1.0**	**N**	**N**	126
**2**	1988	**0.12 M $VOSO_4 \cdot 5H_2O$**	N	H_2SO_4 + MeOH / catechol/methoxide	-1.7 vs. Ag wire	-	**1.5**	**N**	**N**	127
**3**	1993	**Cu**	N	0.2 M $LiClO_4$ in THF : EtOH	< -4.0 vs.AgCl	49	**1.7**	*	**N**	128
**4**	1994	**Cu**	N	0.2 M LiOTF in THF : EtOH	< -4.0 vs.AgCl	60	**2.1**	*	**N**	129
**5**	2016	**Ni**	N	2-propanol H_2O	3.8 V (cell voltage)	0.9	**0.015**	**N**	**N**	130
**6**	2016	**Ni**	Y	0.1 M LiCl in Ethylenediamine	1.8 V (cell voltage)	17	**0.35**	**N**	**N**	131
**7**	2016	**Cp_2TiCl_2**	Y	[C_4mpyr] [eFAP]	-1.5 vs. AgCl	0.2	**69 nmol cm^{-2}**	**N**	**N**	132
**8**	2017	**Fe**	N	[P_{66614}] [eFAP]	-0.8 vs. NHE	60	**0.005**	**Y**	**Y**	133
**9**	2018	**Ag-Au-ZIF**	Y	0.2 M LiOTf in THF:EtOH	-2.9 vs. AgCl	18	**0.01**	**N**	**N**	134
**10**	2018	**Fe-Fe_2O_3**	Y	[C_4mpyr] [eFAP] : FPEE	-0.6 vs. NHE	32	**0.24**	**N**	**Y**	135
**11**	2019	**Cu**	N	0.2 M $LiBF_4$ in THF : EtOH	15 V (cell voltage)	19	**7.9**	*	**N**	136
**12**	2019	**Mo**	N	0.2 M $LiClO_4$ in THF : EtOH	-3.1 vs. AgCl	8	**0.22**	**Y**	**Y**	137
**13**	2020	**Mo**	N	0.5 M $LiClO_4$ in THF : EtOH	-3.8 V vs. Pt pseudo	6	**0.22**	*	**N**	138
**14**	2020	**Cu**	N	0.2 M $LiBF_4$ in THF : EtOH	30 V (cell voltage)	35	**30**	**Y**	**Y**	139
**15**	2020	**Ag-ZIF-Oleylamine**	Y	0.2 M LiOTf in THF : BuOH : H_2O	-2.9 vs. AgCl	18	**0.74 nmol s^{-1} mg^{-1}**	**N**	**N**	140
**16**	2020	**Pt-Au-ZIF**	Y	0.2 M LiOTf in THF : EtOH	-2.9 vs. AgCl	44	**2.6 nmol s^{-1} mg^{-1}**	**N**	**N**	141

Major colour codes are as in Supplementary Table 2

* Relies on relevant and adequate data reported in other published studies on the same system.

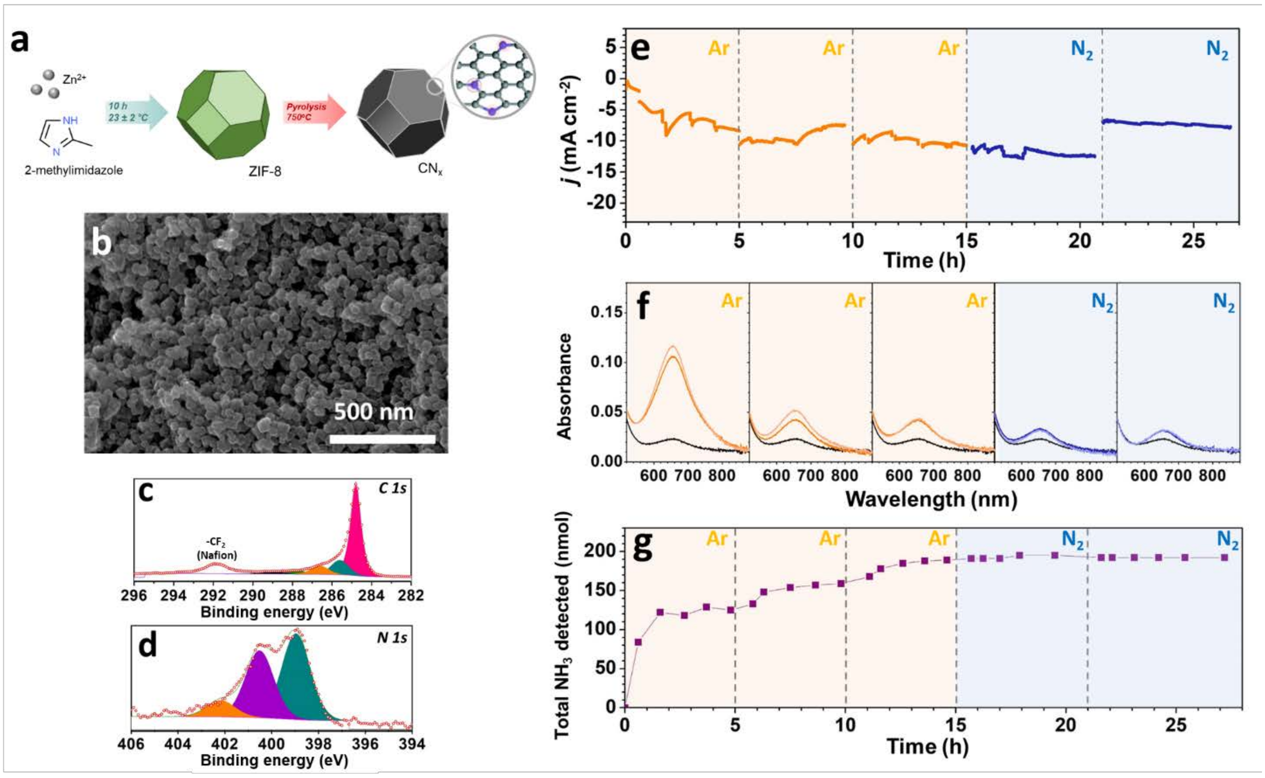

Supplementary Figure 1. Synthesis, characterisation and testing of CN_x materials. **a** Schematic of the synthesis of CN_x. Zeolite imidazole framework-8 was synthesised according to standard literature methods: 2-methylimidazole (3.15 g) and polyvinylpyrrolidone (1.25 g) were dissolved in 50 mL ethanol and vigorously stirred for 1 h under ambient conditions, 3.6 g $Zn(NO_3)_2$ dissolved in 50 ml ethanol was then added dropwise, and the mixture was kept at ambient temperature for *ca* 10 h to allow for the formation of a precipitate, which was collected by centrifugation and washed twice with ethanol (50 mL); dried ZIF-8 was calcined in 5% H_2 in Argon at 750 °C for 5 h (heating ramp 7.5 °C min^{-1}) to produce CN_x. **b** Scanning electron micrograph of CN_x. **c-d** X-ray photoelectron spectroscopic analysis of CN_x: C 1s and N 1s experimental spectra and plausible fitting. The high-resolution N 1s spectrum reveals a total of 4.9 at.% of N-containing functional groups were present. Peaks were fitted with three components assigned to pyridinic-N (*teal*), pyrrolic-N (*purple*), and graphitic-N (*orange*). Particularly, the presence of pyridinic-N, which is described in the literature as the NRR active site, is confirmed by the fitted peak centred at 399.0 eV to be present in atomic abundance of 2.5 at.%. This level of pyridinic N-content is comparable to the amounts reported for the materials in Supplementary Table 1 references 43 and 45. **e** Chronoamperograms recorded with the carbon fibre paper electrode (2.0 cm^2 geometric surface area) modified with 1.2 mg cm^{-2} of CN_x in 0.05 M $H_2SO_{4(aq)}$. A series of sequential tests were undertaken under Ar atmosphere (*orange*) at -0.9 V *vs.* Ag|AgCl|$KCl_{sat.}$; further, the solution was saturated with N_2 and tests were continued at -1.1 V and -0.9 V *vs.* Ag|AgCl|$KCl_{sat.}$ (*blue*). **f** Spectrophotometric detection of ammonia using Berthelot method during tests under Ar (*orange*) and N_2 (*blue*) shown in panel **e** compared to the blank untreated solution (*black*). **g** Amount of NH_3 detected during experiments shown in panel **e**.

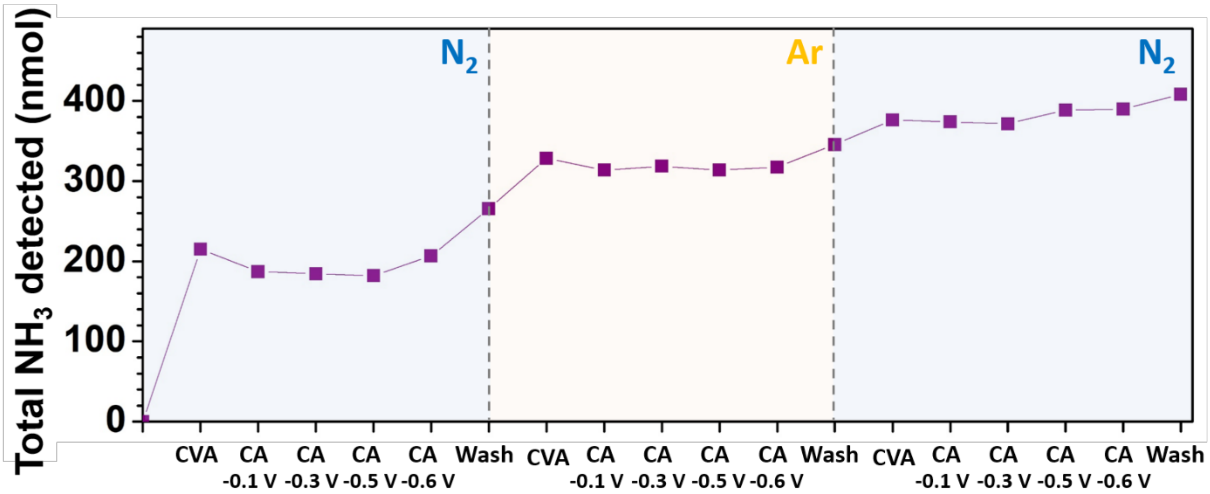

Supplementary Figure 2. Ammonia generation by a vanadium nitride electrode. Total NH_3 produced at each stage of a continuous experiment involving electroreduction of 0.05 M H_2SO_4 aqueous solution with a VN-modified carbon fibre electrode under N_2 (*light blue shading*) and Ar atmosphere (*light yellow shading*). CVA – cyclic voltammetry from 0 to -0.8 V *vs.* RHE at 0.020 V s^{-1}; CA – chronoamperometry for 2 h at a given potential *vs.* RHE; Wash – amount of NH_3 stripped off the electrode using 0.05 M H_2SO_4 solution after electrochemical tests. Further experimental details can be found in [*ACS Sustainable Chem. Eng.* **7**, 6839-6850 (2019); DOI: 10.1021/acssuschemeng.8b06163].

a

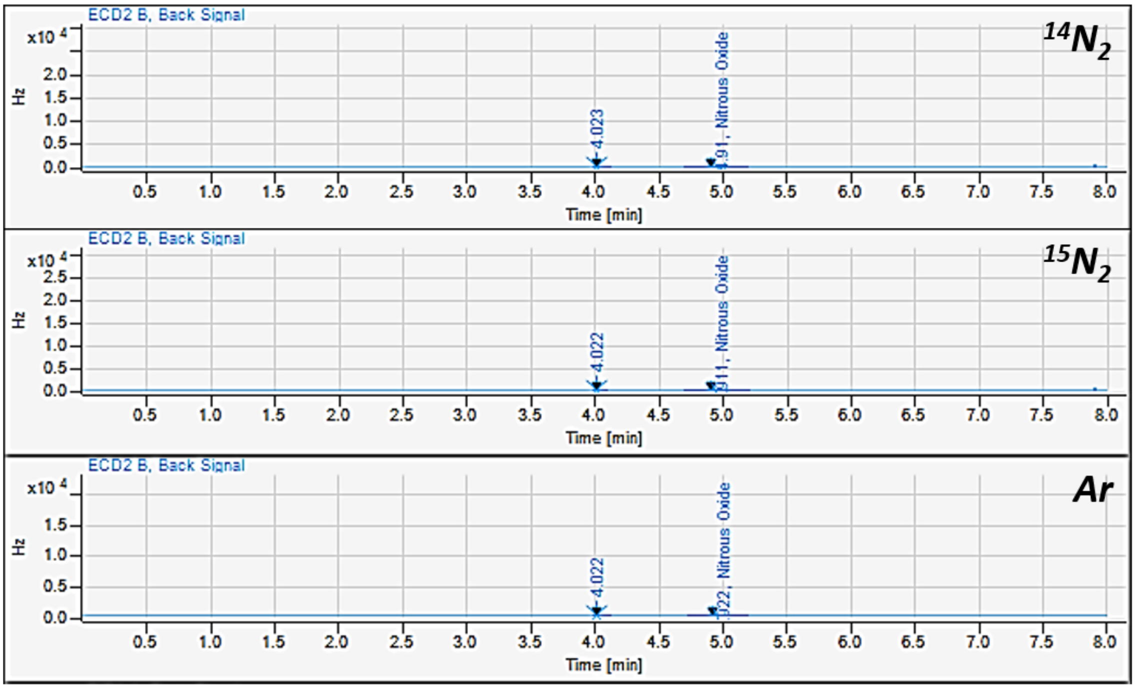

b

Gas	Gas supplier	Purity	Date of gas supply	Date of gas analysis	N_2O concentration
$^{14}N_2$	BOC, Australia	Ultra high purity grade, greater than 99.999%	November 2019	February 2020	0.22 ppm
$^{15}N_2$	Sigma-Aldrich	99.9 % purity (Lot#MBBB3807V)	May 2019	February 2020	0.23 ppm
Ar	BOC, Australia	Ultra high purity grade, greater than 99.999%	September 2019	February 2020	0.17 ppm

Supplementary Figure 3. N_2O content in $^{14}N_2$, $^{15}N_2$ and Ar gas supplies. a Raw unprocessed gas chromatograms for the analysis of N_2O in different gasses; analysis details: Agilent 7890A instrument equipped with an 8 ft. HayeSep Q 80/100 mesh column (Agilent G3591-81047) and an electron coupled detector; N_2 carrier gas; 300 °C detector temperature; 2.5 mL injected volume. Prior to the analysis of gas samples, a standard mixture containing 1 ppm N_2O, 5 ppm CH_4 and 615 ppm CO_2 in Ar was always analysed to confirm the calibration for N_2O. For sampling, each gas was directly purged into a vacuum-evacuated gas-tight syringe; N_2O measurement was then immediately carried out. **b** Summary of the gas specifications and N_2O analysis results.

Supplementary Table 3. Potential NH_3 formation rates from N_2O in flowing-gas and static volume, 4-hour[a] NRR experiments.

Flow rate (mL min^{-1})	N_2 purity[b]	Purging time (min)	Moles N_2[c] (μmol)	Moles N_2O[c] (nmol)	NH_3 from N_2O (nmol s^{-1})
**100**	0.2 ppm N_2O	240	9.84×10^8	1.97×10^2	0.014
**20**	0.2 ppm N_2O	240	1.97×10^8	0.39×10^2	0.003
**0**	0.2 ppm N_2O	15 (prior to experiment at 20 ml min^{-1})	2.23×10^7	0.25×10^1	0.0002

[a] Arbitrarily assumed duration of a typical experiment. [b] The N_2O impurity level in a 99.999% Ultra High Purity N_2 cylinder in our groups was detected by gas chromatography-electron coupled detector (NB: the detected level is well below the manufacturers specifications, further details in Supplementary Figure 3). [c] At 298 K and 1 atm.

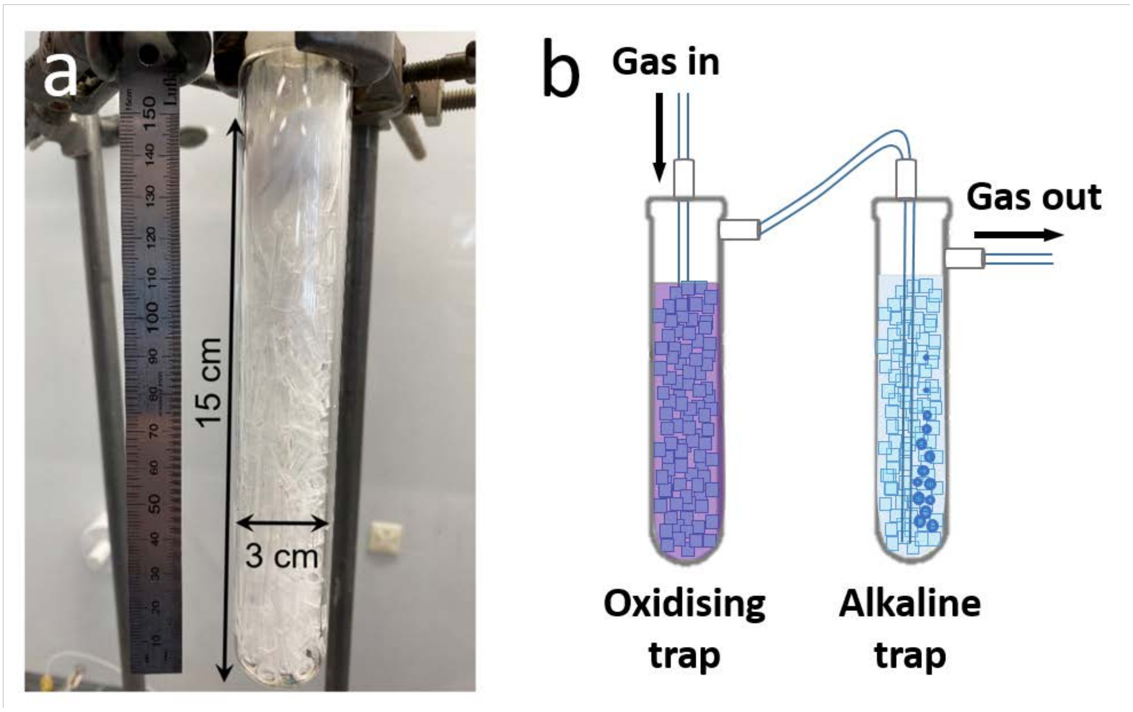

Supplementary Figure 4. Setup for NO_x scrubbing. a Photograph of a body of a trap that is packed with path-blocking materials – glass wool and glass tube pieces. The height of the trap should be as long as possible to create a long, tortuous path for the bubbles through the packing materials. **b** Scheme of the NO_x scrubbing setup with oxidising and alkaline traps connected in series. Any NO_x breakthrough from the oxidising trap (filled with *e.g.* 0.1 M $KMnO_4$ in aqueous 0.1 M KOH) is captured by an alkaline trap (filled with *e.g.* aqueous 0.1 M KOH). Ideally, both traps should include a packing material to create a tortuous pathway for the bubbles, as shown in panel **a**. To determine whether any breakthrough of NO_x species is occurring form the oxidising trap, no increase in NO_3^- and NO_2^- levels over background in the alkaline trap should be regularly confirmed and reported under NRR operating conditions. Otherwise, further improvement of the trap design, in the first place to increase the gas-liquid contact time, is required.

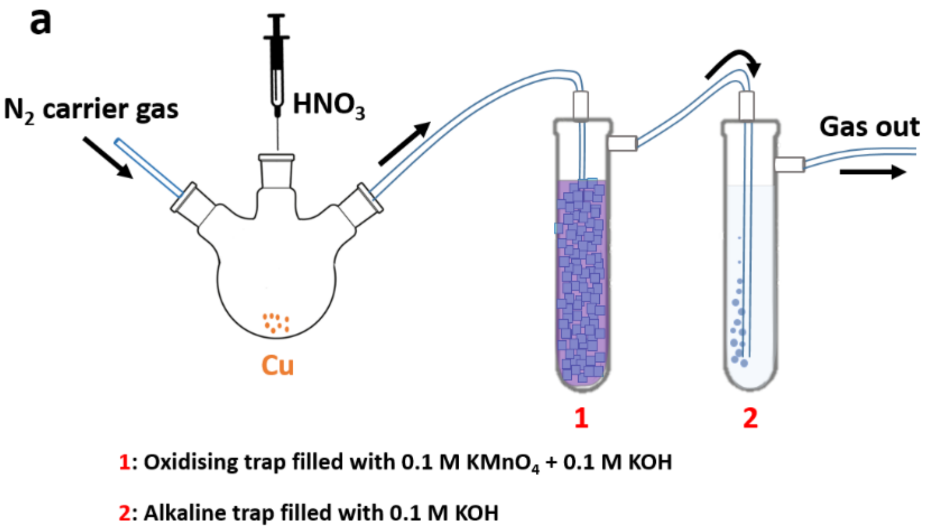

b

Experiment	NO_2 fed through the traps	NO_2^- and NO_3^- in the alkaline trap: Amount	Concentration
**Background**	-	5 nmol (± 2 nmol) NO_2^-<br>47 nmol (± 11 nmol) NO_3^-	1.1 µM (± 0.3 µM)
**#1**	160 µmol	12 nmol NO_2^-<br>64 nmol NO_3^-	1.5 µM
**#2**	160 µmol	12 nmol NO_2^-<br>58 nmol NO_3^-	1.4 µM
**#3**	160 µmol	5 nmol NO_2^-<br>58 nmol NO_3^-	1.3 µM
**Average (#1 – #3)**		10 nmol (± 4 nmol) NO_2^-<br>60 nmol (± 4 nmol) NO_3^-	1.4 µM (± 0.1 µM)

Supplementary Figure 5. Experimental demonstration of NO_x scrubbing. a Schematic diagram of the experimental setup: NO_2 gas was produced by reacting copper powder (80 µmol) and concentrated nitric acid (0.1 mL, *ca* 1.6 mol HNO_3) in a sealed triple-neck round bottom flask installed into the gas supply line prior to the scrubbing setup, which included (1) the oxidising and (2) alkaline traps, as described in Supplementary Figure 4. As soon as all Cu was consumed, the produced NO_2 gas (theoretically, 160 µmol) was introduced into the scrubbing system with an N_2 carrier gas at a flow rate of 20 mL min^{-1}. After 30 min, an aliquot (0.50 mL) was withdrawn from the alkaline trap solution, and the amount of any breakthrough NO_2 species was determined by Griess analysis. **b** Summary of the results for three independent NO_2 scrubbing experiments, each undertaken with fresh solutions in both traps. Background levels of NO_2^- and NO_3^- in 0.1 M KOH trap solution were measured prior to the start of the experiment. Even though a very large amount of NO_2 gas (theoretical 160 µmol) was injected into the system, the NO_x concentration measured in the alkaline scrubber solution remained within the range of the background level.

Supplementary References

1. Köleli F, Kayan DB. Low overpotential reduction of dinitrogen to ammonia in aqueous media. *J. Electroanal. Chem.* **638**, 119-122 (2010).

2. Shi MM*, et al.* Au sub-nanoclusters on TiO2 toward highly efficient and selective electrocatalyst for N2 conversion to NH3 at ambient conditions. *Adv. Mater.* **29**, 1606550 (2017).

3. Bao D*, et al.* Electrochemical reduction of N2 under ambient conditions for artificial N2 fixation and renewable energy storage using N2/NH3 cycle. *Adv. Mater.* **29**, 1604799 (2017).

4. Li SJ, Bao D, Shi MM, Wulan BR, Yan JM, Jiang Q. Amorphizing of Au nanoparticles by CeOx–RGO hybrid support towards highly efficient electrocatalyst for N2 reduction under ambient conditions. *Adv. Mater.* **29**, 1700001 (2017).

5. Kong J*, et al.* Electrochemical synthesis of NH3 at low temperature and atmospheric pressure using a γ-Fe2O3 catalyst. *ACS Sustainable Chem. Eng.* **5**, 10986-10995 (2017).

6. Chen S, Perathoner S, Ampelli C, Mebrahtu C, Su D, Centi G. Room-temperature electrocatalytic synthesis of NH3 from H2O and N2 in a gas–liquid–solid three-phase reactor. *ACS Sustainable Chem. Eng.* **5**, 7393-7400 (2017).

7. Chen S, Perathoner S, Ampelli C, Mebrahtu C, Su D, Centi G. Electrocatalytic synthesis of ammonia at room temperature and atmospheric pressure from water and nitrogen on a carbon-nanotube-based electrocatalyst. *Angew. Chem., Int. Ed.* **56**, 2699-2703 (2017).

8. Chen G-F*, et al.* Ammonia electrosynthesis with high selectivity under ambient conditions via a Li+ incorporation strategy. *J. Am. Chem. Soc.* **139**, 9771-9774 (2017).

9. Yang D, Chen T, Wang Z. Electrochemical reduction of aqueous nitrogen (N 2) at a low overpotential on (110)-oriented Mo nanofilm. *J. Mater. Chem. A* **5**, 18967-18971 (2017).

10. Zhao X, Yin F, Liu N, Li G, Fan T, Chen B. Highly efficient metal–organic-framework catalysts for electrochemical synthesis of ammonia from N 2 (air) and water at low temperature and ambient pressure. *J. Mater. Sci.* **52**, 10175-10185 (2017).

11. Wang D*, et al.* Energy-Efficient Nitrogen Reduction to Ammonia at Low Overpotential in Aqueous Electrolyte under Ambient Conditions. *ChemSusChem* **11**, 3416-3422 (2018).

12. Liu Q*, et al.* Ambient N 2 fixation to NH 3 electrocatalyzed by a spinel Fe 3 O 4 nanorod. *Nanoscale* **10**, 14386-14389 (2018).

13. Manjunatha R, Schechter A. Electrochemical synthesis of ammonia using ruthenium–platinum alloy at ambient pressure and low temperature. *Electrochem. Commun.* **90**, 96-100 (2018).

14. Shi MM, Bao D, Li SJ, Wulan BR, Yan JM, Jiang Q. Anchoring PdCu amorphous nanocluster on graphene for electrochemical reduction of N2 to NH3 under ambient conditions in aqueous solution. *Adv. Energy Mater.* **8**, 1800124 (2018).

15. Wang J, Yu L, Hu L, Chen G, Xin H, Feng X. Ambient ammonia synthesis via palladium-catalyzed electrohydrogenation of dinitrogen at low overpotential. *Nat. Commun.* **9**, 1-7 (2018).

16. Du H, Guo X, Kong R-M, Qu F. Cr 2 O 3 nanofiber: a high-performance electrocatalyst toward artificial N 2 fixation to NH 3 under ambient conditions. *Chem. Commun.* **54**, 12848-12851 (2018).

17. Geng Z*, et al.* Achieving a Record-High Yield Rate of 120.9 for N2 Electrochemical Reduction over Ru Single-Atom Catalysts. *Adv. Mater.* **30**, 1803498 (2018).

18. Han J*, et al.* Ambient N2 fixation to NH3 at ambient conditions: Using Nb2O5 nanofiber as a high-performance electrocatalyst. *Nano Energy* **52**, 264-270 (2018).

19. Hu L, Khaniya A, Wang J, Chen G, Kaden WE, Feng X. Ambient electrochemical ammonia synthesis with high selectivity on Fe/Fe oxide catalyst. *ACS Catal.* **8**, 9312-9319 (2018).

20. Huang H, Xia L, Shi X, Asiri AM, Sun X. Ag nanosheets for efficient electrocatalytic N 2 fixation to NH 3 under ambient conditions. *Chem. Commun.* **54**, 11427-11430 (2018).

21. Li W*, et al.* Nitrogen-free commercial carbon cloth with rich defects for electrocatalytic ammonia synthesis under ambient conditions. *Chem. Commun.* **54**, 11188-11191 (2018).

22. Huang L*, et al.* NbO2 electrocatalyst toward 32% Faradaic efficiency for N2 fixation. *Small Methods* **3**, 1800386 (2019).

23. Li X*, et al.* Enabling Electrocatalytic N2 Reduction to NH3 by Y2O3 Nanosheet under Ambient Conditions. *Ind. Eng. Chem. Res.* **57**, 16622-16627 (2018).

24. Li X*, et al.* Boosted electrocatalytic N2 reduction to NH3 by defect-rich MoS2 nanoflower. *Adv. Energy Mater.* **8**, 1801357 (2018).

25. Qin Q, Heil T, Antonietti M, Oschatz M. Single-Site Gold Catalysts on Hierarchical N-Doped Porous Noble Carbon for Enhanced Electrochemical Reduction of Nitrogen. *Small Methods* **2**, 1800202 (2018).

26. Wang X*, et al.* Atomically dispersed Au1 catalyst towards efficient electrochemical synthesis of ammonia. *Sci. Bull.* **63**, 1246-1253 (2018).

27. Wang Y*, et al.* Rational design of Fe–N/C hybrid for enhanced nitrogen reduction electrocatalysis under ambient conditions in aqueous solution. *ACS Catal.* **9**, 336-344 (2018).

28. Wang Y*, et al.* Boron-doped TiO2 for efficient electrocatalytic N2 fixation to NH3 at ambient conditions. *ACS Sustainable Chem. Eng.* **7**, 117-122 (2018).

29. Qiu W*, et al.* High-performance artificial nitrogen fixation at ambient conditions using a metal-free electrocatalyst. *Nat. Commun.* **9**, 1-8 (2018).

30. Wang Z*, et al.* Ambient electrochemical synthesis of ammonia from nitrogen and water catalyzed by flower-like gold microstructures. *ChemSusChem* **11**, 3480-3485 (2018).

31. Wu X*, et al.* Mn3O4 nanocube: An efficient electrocatalyst toward artificial N2 fixation to NH3. *Small* **14**, 1803111 (2018).

32. Yang X*, et al.* Nitrogen-doped porous carbon: Highly efficient trifunctional electrocatalyst for oxygen reversible catalysis and nitrogen reduction reaction. *J. Mater. Chem. A* **6**, 7762-7769 (2018).

33. Zhang L*, et al.* Ambient NH 3 synthesis via electrochemical reduction of N 2 over cubic sub-micron SnO 2 particles. *Chem. Commun.* **54**, 12966-12969 (2018).

34. Zhang R, *et al.* Enabling effective electrocatalytic N2 conversion to NH3 by the TiO2 nanosheets array under ambient conditions. *ACS Appl. Mater. Interfaces* **10**, 28251-28255 (2018).

35. Yang X, *et al.* Mechanistic insights into electrochemical nitrogen reduction reaction on vanadium nitride nanoparticles. *J. Am. Chem. Soc.* **140**, 13387-13391 (2018).

36. Zhang X, *et al.* TiO 2 nanoparticles–reduced graphene oxide hybrid: an efficient and durable electrocatalyst toward artificial N 2 fixation to NH 3 under ambient conditions. *J. Mater. Chem. A* **6**, 17303-17306 (2018).

37. Zhang Y, *et al.* High-performance electrohydrogenation of N2 to NH3 catalyzed by multishelled hollow Cr2O3 microspheres under ambient conditions. *ACS Catal.* **8**, 8540-8544 (2018).

38. Zhu X, *et al.* Efficient and durable N 2 reduction electrocatalysis under ambient conditions: β-FeOOH nanorods as a non-noble-metal catalyst. *Chem. Commun.* **54**, 11332-11335 (2018).

39. Cui X, Tang C, Liu XM, Wang C, Ma W, Zhang Q. Highly selective electrochemical reduction of dinitrogen to ammonia at ambient temperature and pressure over iron oxide catalysts. *Chem. Eur. J* **24**, 18494-18501 (2018).

40. Han J, *et al.* MoO 3 nanosheets for efficient electrocatalytic N 2 fixation to NH 3. *J. Mater. Chem. A* **6**, 12974-12977 (2018).

41. Yu X, *et al.* Boron-doped graphene for electrocatalytic N2 reduction. *Joule* **2**, 1610-1622 (2018).

42. Liu H-M, *et al.* Surfactant-free atomically ultrathin rhodium nanosheet nanoassemblies for efficient nitrogen electroreduction. *J. Mater. Chem. A* **6**, 3211-3217 (2018).

43. Liu Y, *et al.* Facile ammonia synthesis from electrocatalytic N2 reduction under ambient conditions on N-doped porous carbon. *ACS Catal.* **8**, 1186-1191 (2018).

44. Lv C, *et al.* An Amorphous Noble-Metal-Free Electrocatalyst that Enables Nitrogen Fixation under Ambient Conditions. *Angew. Chem., Int. Ed.* **130**, 6181-6184 (2018).

45. Mukherjee S, *et al.* Metal-organic framework-derived nitrogen-doped highly disordered carbon for electrochemical ammonia synthesis using N2 and H2O in alkaline electrolytes. *Nano Energy* **48**, 217-226 (2018).

46. Cheng H, Ding LX, Chen GF, Zhang L, Xue J, Wang H. Molybdenum carbide nanodots enable efficient electrocatalytic nitrogen fixation under ambient conditions. *Adv. Mater.* **30**, 1803694 (2018).

47. Nazemi M, Panikkanvalappil SR, El-Sayed MA. Enhancing the rate of electrochemical nitrogen reduction reaction for ammonia synthesis under ambient conditions using hollow gold nanocages. *Nano Energy* **49**, 316-323 (2018).

48. Song Y, *et al.* A physical catalyst for the electrolysis of nitrogen to ammonia. *Sci. Adv.* **4**, e1700336 (2018).

49. Zhang L, *et al.* Electrochemical ammonia synthesis via nitrogen reduction reaction on a MoS2 catalyst: theoretical and experimental studies. *Adv. Mater.* **30**, 1800191 (2018).

50. Wang H, *et al.* Ambient electrosynthesis of ammonia: electrode porosity and composition engineering. *Angew. Chem., Int. Ed.* **57**, 12360-12364 (2018).

51. Lv C, *et al.* Defect engineering metal-free polymeric carbon nitride electrocatalyst for effective nitrogen fixation under ambient conditions. *Angew. Chem., Int. Ed.* **130**, 10403-10407 (2018).

52. Ren X, *et al.* Electrochemical N 2 fixation to NH 3 under ambient conditions: Mo 2 N nanorod as a highly efficient and selective catalyst. *Chem. Commun.* **54**, 8474-8477 (2018).

53. Li C, *et al.* Spinel LiMn2O4 nanofiber: an efficient electrocatalyst for N2 reduction to NH3 under ambient conditions. *Inorg. Chem.* **58**, 9597-9601 (2019).

54. Chen P, *et al.* Interfacial engineering of cobalt sulfide/graphene hybrids for highly efficient ammonia electrosynthesis. *Proc. Natl. Acad. Sci. U. S. A.* **116**, 6635-6640 (2019).

55. Han Z, *et al.* Activated TiO2 with tuned vacancy for efficient electrochemical nitrogen reduction. *Appl. Catal., B* **257**, 117896 (2019).

56. Chu K, Liu Y-p, Li Y-b, Zhang H, Tian Y. Efficient electrocatalytic N 2 reduction on CoO quantum dots. *J. Mater. Chem. A* **7**, 4389-4394 (2019).

57. Chu K, Liu Y-p, Wang J, Zhang H. NiO nanodots on graphene for efficient electrochemical N2 reduction to NH3. *ACS Appl. Energy Mater.* **2**, 2288-2295 (2019).

58. Cong L, Yu Z, Liu F, Huang W. Electrochemical synthesis of ammonia from N 2 and H 2 O using a typical non-noble metal carbon-based catalyst under ambient conditions. *Catal. Sci. Technol.* **9**, 1208-1214 (2019).

59. Fan Q, *et al.* High-yield production of few-layer boron nanosheets for efficient electrocatalytic N 2 reduction. *Chem. Commun.* **55**, 4246-4249 (2019).

60. Fu W, Zhuang P, OliverLam Chee M, Dong P, Ye M, Shen J. Oxygen vacancies in Ta2O5 nanorods for highly efficient electrocatalytic N2 reduction to NH3 under ambient conditions. *ACS Sustainable Chem. Eng.* **7**, 9622-9628 (2019).

61. Han L, *et al.* Atomically dispersed molybdenum catalysts for efficient ambient nitrogen fixation. *Angew. Chem., Int. Ed.* **131**, 2343-2347 (2019).

62. He C, *et al.* Identification of FeN4 as an efficient active site for electrochemical N2 reduction. *ACS Catal.* **9**, 7311-7317 (2019).

63. Jin H, *et al.* Nitrogen vacancies on 2D layered W2N3: A stable and efficient active site for nitrogen reduction reaction. *Adv. Mater.* **31**, 1902709 (2019).

64. Kang S, *et al.* Plasma-etching enhanced titanium oxynitride active phase with high oxygen content for ambient electrosynthesis of ammonia. *Electrochem. Commun.* **100**, 90-95 (2019).

65. Kumar RD, *et al.* Trimetallic PdCuIr with long-spined sea-urchin-like morphology for ambient electroreduction of nitrogen to ammonia. *J. Mater. Chem. A* **7**, 3190-3196 (2019).

66. Qin Q, *et al.* Enhanced Electrocatalytic N2 Reduction via Partial Anion Substitution in Titanium Oxide–Carbon Composites. *Angew. Chem., Int. Ed.* **58**, 13101-13106 (2019).

67. Li L, Tang C, Xia B, Jin H, Zheng Y, Qiao S-Z. Two-dimensional mosaic bismuth nanosheets for highly selective ambient electrocatalytic nitrogen reduction. *ACS Catal.* **9**, 2902-2908 (2019).

68. Li X*, et al.* A MoS 2 nanosheet–reduced graphene oxide hybrid: an efficient electrocatalyst for electrocatalytic N 2 reduction to NH 3 under ambient conditions. *J. Mater. Chem. A* **7**, 2524-2528 (2019).

69. Li Y*, et al.* In Situ Growth of Nitrogen-Doped Carbon-Coated γ-Fe2O3 Nanoparticles on Carbon Fabric for Electrochemical N2 Fixation. *ACS Sustainable Chem. Eng.* **7**, 8853-8859 (2019).

70. Liu S, Wang M, Qian T, Ji H, Liu J, Yan C. Facilitating nitrogen accessibility to boron-rich covalent organic frameworks via electrochemical excitation for efficient nitrogen fixation. *Nat. Commun.* **10**, 1-9 (2019).

71. Liu Y*, et al.* Dramatically Enhanced Ambient Ammonia Electrosynthesis Performance by In-Operando Created Li–S Interactions on MoS2 Electrocatalyst. *Adv. Energy Mater.* **9**, 1803935 (2019).

72. Luo Y, Chen G-F, Ding L, Chen X, Ding L-X, Wang H. Efficient electrocatalytic N2 fixation with MXene under ambient conditions. *Joule* **3**, 279-289 (2019).

73. Manjunatha R, Karajić A, Goldstein V, Schechter A. Electrochemical ammonia generation directly from nitrogen and air using an iron-oxide/titania-based catalyst at ambient conditions. *ACS Appl. Mater. Interfaces* **11**, 7981-7989 (2019).

74. Pang F*, et al.* Bimodal nanoporous Pd3Cu1 alloy with restrained hydrogen evolution for stable and high yield electrochemical nitrogen reduction. *Nano Energy* **58**, 834-841 (2019).

75. Ren X*, et al.* High-performance N2-to-NH3 conversion electrocatalyzed by Mo2C nanorod. *ACS Cent. Sci.* **5**, 116-121 (2018).

76. Song P, Wang H, Kang L, Ran B, Song H, Wang R. Electrochemical nitrogen reduction to ammonia at ambient conditions on nitrogen and phosphorus co-doped porous carbon. *Chem. Commun.* **55**, 687-690 (2019).

77. Tao H*, et al.* Nitrogen fixation by Ru single-atom electrocatalytic reduction. *Chem* **5**, 204-214 (2019).

78. Wang F, Liu Yp, Zhang H, Chu K. CuO/graphene nanocomposite for nitrogen reduction reaction. *ChemCatChem* **11**, 1441-1447 (2019).

79. Wang H*, et al.* Direct fabrication of bi-metallic PdRu nanorod assemblies for electrochemical ammonia synthesis. *Nanoscale* **11**, 5499-5505 (2019).

80. Wang H*, et al.* Electrochemical fabrication of porous Au film on Ni foam for nitrogen reduction to ammonia. *Small* **15**, 1804769 (2019).

81. Wang M*, et al.* Over 56.55% Faradaic efficiency of ambient ammonia synthesis enabled by positively shifting the reaction potential. *Nat. Commun.* **10**, 1-8 (2019).

82. Wang Y*, et al.* Generating Defect-Rich Bismuth for Enhancing the Rate of Nitrogen Electroreduction to Ammonia. *Angew. Chem., Int. Ed.* **58**, 9464-9469 (2019).

83. Wang Z*, et al.* Electrocatalytic hydrogenation of N2 to NH3 by MnO: Experimental and theoretical investigations. *Adv. Sci.* **6**, 1801182 (2019).

84. Wu D*, et al.* Ambient electrochemical N 2 reduction to NH 3 under alkaline conditions enabled by a layered K 2 Ti 4 O 9 nanobelt. *Chem. Commun.* **55**, 7546-7549 (2019).

85. Xia L, *et al.* Cr2O3 nanoparticle-reduced graphene oxide hybrid: A highly active electrocatalyst for N2 reduction at ambient conditions. *Inorg. Chem.* **58**, 2257-2260 (2019).

86. Xu B, *et al.* La2O3 nanoplate: An efficient electrocatalyst for artificial N2 fixation to NH3 with excellent selectivity at ambient condition. *Electrochim. Acta* **298**, 106-111 (2019).

87. Yin F, Lin X, He X, Chen B, Li G, Yin H. High Faraday efficiency for electrochemical nitrogen reduction reaction on Co@ N-doped carbon derived from a metal-organic framework under ambient conditions. *Mater. Lett.* **248**, 109-113 (2019).

88. Yu H, *et al.* Bimetallic Ag 3 Cu porous networks for ambient electrolysis of nitrogen to ammonia. *J. Mater. Chem. A* **7**, 12526-12531 (2019).

89. Zhang G, *et al.* Triggering surface oxygen vacancies on atomic layered molybdenum dioxide for a low energy consumption path toward nitrogen fixation. *Nano Energy* **59**, 10-16 (2019).

90. Zhang L, Ding LX, Chen GF, Yang X, Wang H. Ammonia synthesis under ambient conditions: Selective electroreduction of dinitrogen to ammonia on black phosphorus nanosheets. *Angew. Chem., Int. Ed.* **131**, 2638-2642 (2019).

91. Zhang X, *et al.* Boron nanosheet: an elemental two-dimensional (2D) material for ambient electrocatalytic N2-to-NH3 fixation in neutral media. *ACS Catal.* **9**, 4609-4615 (2019).

92. Zhang Y, *et al.* Hexagonal boron nitride nanosheet for effective ambient N 2 fixation to NH 3. *Nano Res.* **12**, 919-924 (2019).

93. Zhao C, *et al.* Ambient electrosynthesis of ammonia on a biomass-derived nitrogen-doped porous carbon electrocatalyst: Contribution of pyridinic nitrogen. *ACS Energy Lett.* **4**, 377-383 (2019).

94. Zhao J, *et al.* Defect-rich fluorographene nanosheets for artificial N 2 fixation under ambient conditions. *Chem. Commun.* **55**, 4266-4269 (2019).

95. Zheng J, *et al.* Tuning the Electron Localization of Gold Enables the Control of Nitrogen-to-Ammonia Fixation. *Angew. Chem., Int. Ed.* **58**, 18604-18609 (2019).

96. Xue Z-H, *et al.* Electrochemical reduction of N2 into NH3 by donor–acceptor couples of Ni and Au nanoparticles with a 67.8% Faradaic efficiency. *J. Am. Chem. Soc.* **141**, 14976-14980 (2019).

97. Hao Y-C, *et al.* Promoting nitrogen electroreduction to ammonia with bismuth nanocrystals and potassium cations in water. *Nat. Catal.* **2**, 448-456 (2019).

98. Liu YT, Chen X, Yu J, Ding B. Carbon-Nanoplated CoS@ TiO2 Nanofibrous Membrane: An Interface-Engineered Heterojunction for High-Efficiency Electrocatalytic Nitrogen Reduction. *Angew. Chem., Int. Ed.* **58**, 18903-18907 (2019).

99. Wang Xh, Wang J, Li Yb, Chu K. Nitrogen-Doped NiO Nanosheet Array for Boosted Electrocatalytic N2 Reduction. *ChemCatChem* **11**, 4529-4536 (2019).

100. Cheng H, Cui P, Wang F, Ding LX, Wang H. High efficiency electrochemical nitrogen fixation achieved with a lower pressure reaction system by changing the chemical equilibrium. *Angew. Chem., Int. Ed.* **58**, 15541-15547 (2019).

101. Chen X, Liu Y-T, Ma C, Yu J, Ding B. Self-organized growth of flower-like SnS 2 and forest-like ZnS nanoarrays on nickel foam for synergistic superiority in electrochemical ammonia synthesis. *J. Mater. Chem. A* **7**, 22235-22241 (2019).

102. Zhao X, *et al.* In situ nano Au triggered by a metal boron organic polymer: efficient electrochemical N 2 fixation to NH 3 under ambient conditions. *J. Mater. Chem. A* **7**, 20945-20951 (2019).

103. Zang W, *et al.* Copper single atoms anchored in porous nitrogen-doped carbon as efficient pH-Universal catalysts for the nitrogen reduction reaction. *ACS Catal.* **9**, 10166-10173 (2019).

104. Guo J, *et al.* Tunable synthesis of multiply twinned intermetallic Pd 3 Pb nanowire networks toward efficient N 2 to NH 3 conversion. *J. Mater. Chem. A* **7**, 20247-20253 (2019).

105. Zou H, Rong W, Long B, Ji Y, Duan L. Corrosion-Induced Cl-Doped Ultrathin Graphdiyne toward Electrocatalytic Nitrogen Reduction at Ambient Conditions. *ACS Catal.* **9**, 10649-10655 (2019).

106. Li C, Fu Y, Wu Z, Xia J, Wang X. Sandwich-like reduced graphene oxide/yolk–shell-structured Fe@ Fe 3 O 4/carbonized paper as an efficient freestanding electrode for electrochemical synthesis of ammonia directly from H 2 O and nitrogen. *Nanoscale* **11**, 12997-13006 (2019).

107. Ahmed MI, Chen S, Ren W, Chen X, Zhao C. Synergistic bimetallic CoFe 2 O 4 clusters supported on graphene for ambient electrocatalytic reduction of nitrogen to ammonia. *Chem. Commun.* **55**, 12184-12187 (2019).

108. Lin Y-X, *et al.* Boosting selective nitrogen reduction to ammonia on electron-deficient copper nanoparticles. *Nat. Commun.* **10**, 1-7 (2019).

109. Zhao J, *et al.* Efficient electrohydrogenation of N 2 to NH 3 by oxidized carbon nanotubes under ambient conditions. *Chem. Commun.* **55**, 4997-5000 (2019).

110. Tong W, Huang B, Wang P, Li L, Shao Q, Huang X. Crystal-Phase-Engineered PdCu Electrocatalyst for Enhanced Ammonia Synthesis. *Angew. Chem., Int. Ed.* **59**, 2649-2653 (2020).

111. Huang H, Li F, Xue Q, Zhang Y, Yin S, Chen Y. Salt-Templated Construction of Ultrathin Cobalt Doped Iron Thiophosphite Nanosheets toward Electrochemical Ammonia Synthesis. *Small* **15**, 1903500 (2019).

112. Tan L, *et al.* Synthesis of ammonia via electrochemical nitrogen reduction on high-index faceted Au nanoparticles with a high faradaic efficiency. *Chem. Commun.* **55**, 14482-14485 (2019).

113. Chen Y, Wu B, Sun B, Wang N, Hu W, Komarneni S. N-doped porous carbon self-generated on nickel oxide nanosheets for electrocatalytic N2 fixation with a Faradaic efficiency beyond 30%. *ACS Sustainable Chem. Eng.* **7**, 18874-18883 (2019).

114. Peng M, *et al.* Bioinspired Fe3C@ C as Highly Efficient Electrocatalyst for Nitrogen Reduction Reaction under Ambient Conditions. *ACS Appl. Mater. Interfaces* **11**, 40062-40068 (2019).

115. Wu T, *et al.* Greatly improving electrochemical N2 reduction over TiO2 nanoparticles by iron doping. *Angew. Chem., Int. Ed.* **58**, 18449-18453 (2019).

116. Liu YT, Li D, Yu J, Ding B. Stable Confinement of Black Phosphorus Quantum Dots on Black Tin Oxide Nanotubes: A Robust, Double-Active Electrocatalyst toward Efficient Nitrogen Fixation. *Angew. Chem., Int. Ed.* **131**, 16591-16596 (2019).

117. Suryanto BH, *et al.* MoS2 polymorphic engineering enhances selectivity in the electrochemical reduction of nitrogen to ammonia. *ACS Energy Lett.* **4**, 430-435 (2018).

118. Wang Y, *et al.* Self-supported NbSe2 nanosheet arrays for highly efficient ammonia electrosynthesis under ambient conditions. *J. Catal.* **381**, 78-83 (2020).

119. Zhang J, Ji Y, Wang P, Shao Q, Li Y, Huang X. Adsorbing and Activating N2 on Heterogeneous Au–Fe3O4 Nanoparticles for N2 Fixation. *Adv. Funct. Mater.* **30**, 1906579 (2020).

120. Wang J, *et al.* A General Strategy to Glassy M-Te (M= Ru, Rh, Ir) Porous Nanorods for Efficient Electrochemical N2 Fixation. *Adv. Mater.* **32**, 1907112 (2020).

121. Ren J-T, Wan C-Y, Pei T-Y, Lv X-W, Yuan Z-Y. Promotion of electrocatalytic nitrogen reduction reaction on N-doped porous carbon with secondary heteroatoms. *Appl. Catal., B* **266**, 118633 (2020).

122. Wang H-B, *et al.* Bionic Design of a Mo (IV)-Doped FeS2 Catalyst for Electroreduction of Dinitrogen to Ammonia. *ACS Catal.* **10**, 4914-4921 (2020).

123. Tong Y, *et al.* Vacancy Engineering of Iron-Doped W18O49 Nanoreactors for Low-Barrier Electrochemical Nitrogen Reduction. *Angew. Chem., Int. Ed.* **59**, 7356-7361 (2020).

124. Peng G, Wu J, Wang M, Niklas J, Zhou H, Liu C. Nitrogen-Defective Polymeric Carbon Nitride Nanolayer Enabled Efficient Electrocatalytic Nitrogen Reduction with High Faradaic Efficiency. *Nano Letters* **20**, 2879-2885 (2020).

125. Zhang L, *et al.* A Janus Fe-SnO2 Catalyst that Enables Bifunctional Electrochemical Nitrogen Fixation. *Angew. Chem., Int. Ed.* **132**, 10980-10985 (2020).

126. Becker JY, Avraham S, Posin B. Nitrogen fixation: Part I. Electrochemical reduction of titanium compounds in the presence of catechol and N2 in MeOH or THF. *J. Electroanal. Chem. Interfacial Electrochem.* **230**, 143-153 (1987).

127. Becker JY, Posin B. Nitrogen fixation: Part II. Nitrogen reduction by electrochemically generated vanadium (II) promoted by various organic ligands in basic methanol. *J. Electroanal. Chem. Interfacial Electrochem.* **250**, 385-397 (1988).

128. Tsuneto A, Kudo A, Sakata T. Efficient electrochemical reduction of N2 to NH3 catalyzed by lithium. *Chem. Lett.* **22**, 851-854 (1993).

129. Tsuneto A, Kudo A, Sakata T. Lithium-mediated electrochemical reduction of high pressure N2 to NH3. *J. Electroanal. Chem.* **367**, 183-188 (1994).

130. Kim K, Lee N, Yoo C-Y, Kim J-N, Yoon HC, Han J-I. Communication—electrochemical reduction of nitrogen to ammonia in 2-propanol under ambient temperature and pressure. *J. Electrochem. Soc.* **163**, F610 (2016).

131. Kim K, Yoo C-Y, Kim J-N, Yoon HC, Han J-I. Electrochemical synthesis of ammonia from water and nitrogen in ethylenediamine under ambient temperature and pressure. *J. Electrochem. Soc.* **163**, F1523 (2016).

132. Katayama A, Inomata T, Ozawa T, Masuda H. Electrochemical conversion of dinitrogen to ammonia induced by a metal complex–supported ionic liquid. *Electrochem. Commun.* **67**, 6-10 (2016).

133. Zhou F*, et al.* Electro-synthesis of ammonia from nitrogen at ambient temperature and pressure in ionic liquids. *Energy Environ. Sci.* **10**, 2516-2520 (2017).

134. Lee HK*, et al.* Favoring the unfavored: selective electrochemical nitrogen fixation using a reticular chemistry approach. *Sci. Adv.* **4**, eaar3208 (2018).

135. Suryanto BH*, et al.* Rational electrode–electrolyte design for efficient ammonia electrosynthesis under ambient conditions. *ACS Energy Lett.* **3**, 1219-1224 (2018).

136. Lazouski N, Schiffer ZJ, Williams K, Manthiram K. Understanding continuous lithium-mediated electrochemical nitrogen reduction. *Joule* **3**, 1127-1139 (2019).

137. Andersen SZ*, et al.* A rigorous electrochemical ammonia synthesis protocol with quantitative isotope measurements. *Nature* **570**, 504-508 (2019).

138. Schwalbe JA*, et al.* A Combined Theory-Experiment Analysis of the Surface Species in Lithium-Mediated NH3 Electrosynthesis. *ChemElectroChem* **7**, 1542-1549 (2020).

139. Lazouski N, Chung M, Williams K, Gala ML, Manthiram K. Non-aqueous gas diffusion electrodes for rapid ammonia synthesis from nitrogen and water-splitting-derived hydrogen. *Nat. Catal.* **3**, 463-469 (2020).

140. Koh CSL, Lee HK, Fan Sim HY, Han X, Phan-Quang GC, Ling XY. Turning Water from a Hindrance to the Promotor of Preferential Electrochemical Nitrogen Reduction. *Chem. Mater.* **32**, 1674-1683 (2020).

141. Sim HYF*, et al.* ZIF-induced d-band Modification in Bimetallic Nanocatalyst: Achieving> 44% Efficiency in Ambient Nitrogen Reduction Reaction. *Angew. Chem., Int. Ed.* **132**, 17145-17151 (2020).
